# The ‘healthy = sustainable’ heuristic: Effects of health and sustainability labels on perceived sustainability and healthiness of foods

**DOI:** 10.1111/aphw.70031

**Published:** 2025-05-07

**Authors:** Katharina N. Eichin, Agnes Effert, Britta Renner, Gudrun Sproesser

**Affiliations:** ^1^ Johannes Kepler Universität Linz Austria; ^2^ Universität Konstanz Constance Germany

**Keywords:** food halo, food heuristic, food labelling, food sustainability, the ‘healthy = sustainable’ heuristic

## Abstract

Studies show that foods perceived as healthier are often also seen as more sustainable, suggesting a ‘healthy = sustainable’ heuristic. However, the direction of this effect remains unclear. This study aims to investigate (1) whether perceived healthiness influences perceived sustainability or whether the reverse effect occurs and (2) whether inter‐individual differences, such as health interest, moderate these effects.

In an online study, participants (n = 712) were shown pictures of foods with high or low healthiness indices and asked to rate their sustainability. Conversely, they were also shown pictures of foods with high or low sustainability indices and asked to rate their healthiness. Additionally, participants' interest in health and sustainability and their belief in a relationship between the healthiness and sustainability of foods were measured. Exploratory analyses included label credibility as a control variable.

Foods with a higher healthiness index were perceived as more sustainable (effect size: δ = 0.39; [CI: 0.36; 0.41]). Similarly, higher sustainability indices led to higher healthiness ratings (effect size: δ = 0.22; [CI: 0.20; 0.24]). Moderating effects of interests were small and dependent on whether label credibility was accounted for.

The results support the assumption of a ‘healthy = sustainable’ heuristic, indicating that the effect is bidirectional. The implications for food labelling are discussed.

## INTRODUCTION

Healthy eating has increased in importance to consumers in the last few years (Grimmelt et al., 2022). Individuals are interested in healthy diets as a means to control their weight and decrease the risk of non‐communicable diseases (e.g., cardiovascular disease; Dijkstra et al., 2014) but also to improve their general health and body image (Ashton et al., 2017). This growing demand is also reflected in a high number of social media channels advertising the benefits of healthy lifestyles (Bak & Priniski, 2020). A newer development is the increasing interest in sustainability topics: climate activism has become more present (de Moor et al., 2021), eating behaviour has been identified as an important contribution to planetary health (Willett et al., 2019) and, importantly the United Nations (UN) has formulated goals for sustainable development that also include sustainable food consumption (United Nations, 2015). While these developments indicate a growing interest in these topics and individuals have a general understanding of what healthy eating entails (Gruber et al., 2022), knowledge about which foods are sustainable is often insufficient (Kenny et al., 2023). According to the UN, sustainable food consumption includes among others the dimensions of low environmental impact, e.g., the use of energy, water and other natural resources, the efficiency in production and packaging also being beneficial for the individual and society (Food and Agricultural Organization of the United Nations & World Health Organization, 2019; Renner et al., 2021). This multidimensionality makes it rather complex to evaluate options.

Dual‐process theories suggest that there are two different cognitive systems humans use to make decisions. System 1 on the one hand operates in a fast and automatic way and requires little effort. System 2 on the other hand is more controlled and analytic but also requires more time and effort (see e.g., Evans, 2008). Where the complexity of properly evaluating food options would require the slower type of system 2 processing, this is at odds with how food choices are made in daily life. Decisions in places like supermarkets or canteens often need to be taken quickly, preventing thorough consideration of all relevant aspects of a product or meal. In such situations, individuals often resort to heuristic processes belonging to system 1 to evaluate their options in a fast and efficient manner (Gigerenzer & Gaissmaier, 2011). Generally, heuristic processing is a useful strategy for reducing the complexity of a problem. However, it can sometimes result in erroneous decisions (Tversky & Kahneman, 1974).

### The ‘healthy = sustainable’ heuristic

Interestingly, individuals seem to rely on heuristic processes when evaluating the healthiness and sustainability of foods. When people are asked to rate these aspects, the two dimensions are correlated: foods perceived as being healthier are also seen as more sustainable, and those perceived as being unhealthier are also seen as less sustainable (Lazzarini et al., 2016; Sproesser, Arens‐Azevedo, & Renner, 2023). It has therefore been suggested that individuals apply a ‘healthy = sustainable’ heuristic to make these judgements (see Sproesser, Arens‐Azevedo, & Renner, 2023). While the use of heuristics can generally be adaptive (see e.g., Gigerenzer & Gaissmaier, 2011) and the perceived positive relationship between healthiness and sustainability can be accurate in some instances (e.g., many vegetables or processed red meat), this is not always the case (e.g., air‐transported fruits). Consequently, relying on a ‘healthy = sustainable’ heuristic’ to assess food properties can be misleading.

### Food halos

While the correlation between perceived healthiness and sustainability of foods has been observed repeatedly (Lazzarini et al., 2016; Sproesser, Arens‐Azevedo, & Renner, 2023; Verain et al., 2016), the origin of this relationship remains unclear. Do healthiness perceptions influence sustainability judgements, or is the reverse true? Alternatively, could a third variable be influencing judgements on both dimensions? A possible mechanism to explain the relationship is the so‐called halo effect (Thorndike, 1920) which describes a cognitive bias where a favourable impression in one domain (e.g., packaging, labels) extends to other unrelated domains (Schuldt et al., 2012). This can make a food appear healthier than it actually is (see e.g., Besson et al., 2019). Conversely, perceiving something as healthy can also influence its evaluation on other dimensions.

Previous literature has identified several food‐related attributes to induce halo effects. For instance, organic, fair trade and vegetarian or vegan labels have been found to increase healthiness perceptions. Foods with organic labels are often perceived as healthier (Nadricka et al., 2020), lower in calorie content (Besson et al., 2019) and lower in fat content (Lee et al., 2013). This effect even extends to low‐healthiness snack foods; for example, organic chocolate cookies are perceived as lower in calorie content compared to their non‐organic counterparts (Richetin et al., 2022), and organic chips were seen as lower in fat and higher in fibre (Lee et al., 2013). Organic labels are not the only type of food labels that enhance healthiness perceptions. Fair trade labelling has also been shown to affect food perceptions beyond social responsibility and increase consumption by boosting perceived healthiness (Berry & Romero, 2021). Similar effects have been found for foods labelled as vegetarian (Besson et al., 2019) and vegan (Bullock et al., 2020). These findings suggest that beliefs about aspects of sustainability (i.e., organic, fair‐trade) can influence healthiness perceptions. However, this relationship is not uniformly consistent. Bschaden et al. (2022) conducted a study where participants were invited to either a ‘snack tasting’ or a ‘tasting of a sustainable snack’. In the latter condition, participants were informed that the snack was produced from by‐products that would otherwise be treated as food waste. This information did not induce a halo effect on the perceived healthiness of the snack. To our knowledge, no study has investigated the effect of actual sustainability labelling on healthiness perceptions.

While the above studies focus on factors influencing healthiness perceptions, the reverse can also occur: healthiness perceptions can influence judgements of other food attributes. For example, sandwiches that appeared healthier but had the same actual calorie content were rated as having a lower calorie content (Chandon & Wansink, 2007). Labelling food as healthy can also increase its perceived tastiness in some cultures (Werle et al., 2013) while decreasing it in others (Raghunathan et al., 2006). However, there are no studies investigating whether perceived healthiness influences perceived sustainability. Hence, the first aim of this study was to investigate whether healthiness perceptions affect sustainability judgements, whether sustainability perceptions affect healthiness judgements, or whether both is the case.

If the ‘healthy = sustainable’ heuristic stems from a bidirectional relationship between perceived healthiness and perceived sustainability, the follow‐up question is whether one of these effects is stronger. It is plausible that the effect of perceived healthiness on sustainability judgements is stronger than the reverse. Specifically, when individuals were asked to assemble an environmentally friendly meal, they chose fewer grams of dessert compared to a control group that assembled a meal to their liking (Wassmann et al., 2023). This suggests that participants might have used healthiness as a cue for sustainability. Sproesser, Aulbach, et al. (2023) also argued that individuals might be more familiar with indicators of food healthiness compared to food sustainability. Consequently, they might be more likely to use healthiness as a basis for their judgements.

### The moderating effects of interests and beliefs on food halos

The strength of food halo effects can differ between individuals depending on their interests and beliefs. Several studies have examined the moderating effects of interests related to the attribute producing the halo effect, such as environmental interest, when investigating the effects of organic labels. For instance, individuals with higher pro‐environmentalism showed stronger halo effects in response to organic claims (Schuldt & Schwarz, 2010) and eco‐labels (Sörqvist et al., 2015; Vecchio et al., 2017). However, while more evidence points towards larger halo effects of eco‐labels on estimated health dimensions in people with higher environmental interest, some studies found no moderating effect or even the reverse pattern (see Apaolaza et al., 2017; Lee et al., 2013). This conflicting evidence might partly stem from differences in the conceptualization of ‘environmental interest’, with the studies finding larger halo effects in people with higher environmental interest focusing more often on attitudinal as opposed to behavioural aspects. Given this context, a second aim of this study was to investigate whether attitudinal aspects of interests related to the label topic (i.e., health and sustainability) increase the label‐induced halo effects on food ratings.

There is also the possibility that interests related to the *outcome* variable (i.e., food ratings) produce moderating effects. Apaolaza et al. (2017) found that the halo effect of organic labels on health perceptions of wine was reduced in health‐concerned individuals. However, the evidence for this is very limited. Therefore, a third aim of the study was to investigate the moderating effect of interests related to the outcome variable regarding the label‐induced halo effects on food ratings.

In addition to interests, an explicit belief in a relationship between healthiness and sustainability might be important for the halo effect. Previous studies found that the ‘unhealthy = tasty’ heuristic was larger the more participants agreed with statements such as “There is no way to make food healthier without sacrificing taste” (Haasova & Florack, 2019; Raghunathan et al., 2006). However, as of yet, there is no study on the effect of an explicit belief in a relationship between healthiness and sustainability on halo effects regarding health and sustainability. A fourth aim of the present study was therefore to investigate the influence of the explicit belief in a relationship between healthiness and sustainability on potential halo effects.

### The present study

The present study aimed to examine the directionality of the ‘healthy = sustainable’ heuristic and identify potential influencing factors. Specifically, the first aim was to investigate whether there is a halo effect from healthiness towards sustainability or vice versa. The second and third aims were to examine the moderating role of interests related both to the halo‐effect producing attribute and to the outcome rating. Finally, the fourth aim was to investigate the moderating role of the explicit belief in a relationship between healthiness and sustainability.

In an online experiment, participants viewed images of foods labelled with either a healthiness or a sustainability score. Perceived healthiness was manipulated by presenting the same foods with either a low healthiness score or with a high healthiness score. Similarly, perceived sustainability was manipulated by showing the foods with either low or high sustainability scores.

### Hypotheses

We had the following preregistered hypotheses (Eichin et al., 2023):

Individuals rate the *sustainability* of foods higher in condition with high healthiness scores compared to low healthiness scores.

The effect of healthiness scores on sustainability ratings is larger for individuals with a higher *interest in health*.

The effect of healthiness scores on sustainability ratings is influenced by individuals' *interest in sustainability*.

The effect of healthiness scores on sustainability ratings is higher for individuals with a stronger individual *belief that healthiness equals sustainability*.

Individuals rate the *healthiness* of foods higher in the condition with high sustainability scores compared to low sustainability scores.

The effect of sustainability scores on healthiness ratings is influenced by individuals' *interest in health*.

The effect of sustainability scores on healthiness ratings is higher for individuals with a higher *interest in sustainability*.

The effect of sustainability labels on healthiness ratings is higher for individuals with a stronger individual *belief that healthiness equals sustainability*.

The effect of healthiness scores on sustainability ratings is larger than the effect of sustainability scores on healthiness ratings.

## METHODS

### Ethics statement

Participants were treated in accordance with the declaration of Helsinki. The study was approved by the Ethics Committee of Johannes Kepler University Linz.

### Experimental design

In a 2 × 2 mixed design online study, participants saw eight food images. Four images were labelled with a *sustainability* score and four images were labelled with a *healthiness* score (within‐factor label type). The label scores were experimentally manipulated between groups (between‐factor label score), such that the same foods appeared with a low score for some participants and with a high score for others. Participants were randomly assigned to one of four conditions: high‐healthiness and high‐sustainability labels, high‐healthiness and low‐sustainability labels, low‐healthiness and high‐sustainability labels, low‐healthiness and low‐sustainability labels. The reason for the between‐subject design was to be able to compare the same foods with different labels.

### Pre‐test image selection and label design

**FIGURE 1:**
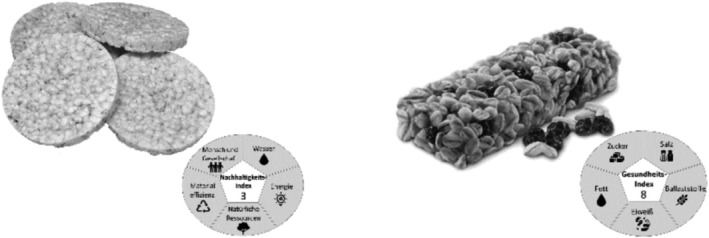
Examples of labelled food images. *Note*. Images are depicted in greyscale but were presented in colour in the study. The image on the left shows an example of a labelled food image with a sustainability manipulation. The German text translates to ‘sustainability index’ in the Centre of the label, surrounded by ‘water’, ‘energy’, ‘natural resources’, ‘material efficiency’ and ‘People and Society’. The image on the right shows an example of a labelled food image with a healthiness manipulation. The German text translates to ‘health index’ in the Centre of the label, surrounded by ‘salt’, ‘fibre’, ‘protein’, ‘fat’ and ‘sugar’.

Images were retrieved from food image databases (Blechert et al., 2014; Toet et al., 2019) and an online image database (chandlervid85 from flaticon.com, n.d.). To select images for the main study, a convenience sample of *N* = 24 participants (mostly university students and female) rated the healthiness and sustainability of 34 food images on a scale from 1 to 10. This was necessary because the databases did not include ratings on perceived sustainability. To ensure that all images could be presented credibly for the different conditions in the main study, only images that were neither particularly healthy nor sustainable were included. In total, eight images (snacks, breakfast foods, meals) with average ratings between 4 and 6 for both healthiness and sustainability were selected. All foods were processed (e.g., salted peanuts, rice cakes) or ultra‐processed (e.g., bagels, cereal bars) with the same ratio across conditions. Of these eight selected stimuli, four were assigned to the healthiness condition (low/high healthiness score) and four to the sustainability condition (low/high sustainability score). Low scores ranged between 2 and 4 and high scores between 7 and 9. The design of labels was adapted from Cho and Baskin (2018) and consisted of a numeric score indicating healthiness or sustainability surrounded by relevant subdomains of the respective concepts. For the healthiness labels, the subdomains were sugar, fat, protein, salt and dietary fibre; for the sustainability label, the subdomains were people and society, water, energy, natural resources and efficiency of materials. Example images are displayed in Figure 1; for an overview of all images see the supplement.

### Power considerations

Power was calculated using the InteractionPoweR package in R (Green & MacLeod, 2016). Assuming a medium effect for the effect of the label score and small effects for the moderating variables as well as the interaction between label scores and moderating variables, a sample size of N = 710 was determined. Please note that, due to the complexity of power analysis in the Bayesian framework, power was calculated with a frequentist approach.

### Data collection and exclusion criteria

Participants were recruited and compensated via a commercial panel provider (‘Cint’). We set quotas for gender, age, educational level and region to obtain a sample representative for the Austrian population. Participants were required to be at least 18 years old and reside in Austria. The survey included two attention checks. For attention, check 1, participants were asked to ‘select 8’ in a list of food ratings. For attention check 2, participants were asked to select ‘slightly agree’ on a questionnaire item. Participants were excluded if they failed one of the two attention checks (*n* = 1,455), if they completed the survey in less than 50% of the median response time (*n* = 31) and if they gave the same answer to all items displayed on the same page i.e., their answering pattern equalled a straight line, on at least five out of eleven pages (*n* = 16). We chose this criterium to account for the possibility that individuals might sometimes purposefully give the same rating for all items on a page (e.g., rating a food as five on health, sustainability, taste, etc.). Showing this answering pattern across many pages, however, is more likely to indicate straight lining, i.e., the attempt to get through the survey quickly. The final sample consisted of *N* = 712 participants. To avoid missing data all survey responses were set to mandatory, i.e., the survey could not be completed if an answer was missing.

### Procedure

Participants were informed about the content of the study and agreed to participate. As information about the study's hypotheses might have influenced participants' judgements, they received the broad information that the purpose of the study was to investigate how consumers evaluate healthiness and sustainability labels on foods as well as to investigate how consumers rated certain foods on different attributes (e.g., healthiness, liking, etc.). After completing demographic questions, they received information about the study tasks. To familiarize them with the two different label types and the interpretation of the label scores, they first rated the labels on study‐unrelated criteria (e.g., appearance). Then, in the main part of the study, they viewed and rated the foods labelled with the healthiness or sustainability scores and filled in questionnaires concerning their interest in health and sustainability, as well as their belief in the relationship between healthiness and sustainability, i.e., that foods that are sustainable are also healthy and vice versa. Last, they rated the credibility of the labels on a scale from 1 (not at all) to 10 (totally). After completion of all tasks, participants were informed that label scores were not realistic reflections of the foods' healthiness and sustainability and received full disclosure about the study's purposes.

### Questionnaires

Interest in healthy eating was measured with the eight‐item general health interest scale (Roininen et al., 1999) which was translated to German and back‐translated to ensure accuracy. An example item is ‘The healthiness of snacks makes no difference to me’. The internal consistency in this sample was α = .87.

Interest in sustainable eating was measured using an adapted version of the eight‐item general health interest scale (Roininen et al., 1999), replacing health‐related words by sustainability‐related words (for details see supplement). An example item is ‘The sustainability of snacks makes no difference to me’. The internal consistency in this sample was α = .90.

To measure the strength of the belief in a relationship between healthiness and sustainability of foods we used a three‐item scale (e.g., ‘Foods that are good for my health are also good for the environment’; for the full scale see supplement). The internal consistency in this sample was α = .82.

### Data analysis

Data were analysed using R (R Core Team, 2022) and RStudio (Posit team, 2023). To analyse the effect of healthiness and sustainability labels (high vs. low label scores) on food ratings, Bayesian t‐tests were conducted using the bayest package, estimating the effect size δ of the difference (Kelter, 2020). The effect is considered small when δ lies between 0.2 and 0.5, medium for 0.5 and 0.8 and large for δ larger than 0.8 (Cohen, 2009). To control if the effect of labels differed between food types, ANOVAs comparing the effect of health/sustainability scores on ratings between different food types were calculated. ANOVAs were calculated using the rstatix package in R (Kassambara, 2021). There were no significant effects of food type, however, there was a tendency for one food to show lower health score effects. For details see supplement Tables S1–S3. For the analyses of the moderating effects of person‐level variables (interest in health and sustainability and the belief in a relationship between the healthiness and sustainability of foods) on the relationship between food labels and ratings, Bayesian linear models were conducted using brms (Bürkner, 2017) for each moderator separately. As additional non‐preregistered analyses, we repeated all analyses controlling for label credibility. For the effects of labels on food ratings, separate t‐tests were conducted for high and low label credibility. Low‐label credibility comprised ratings on the lower half of the scale (1–5); conversely high‐label credibility comprised ratings on the upper half of the scale (6–10). For the moderation analyses, credibility ratings were included as a control variable. All credibility analyses were conducted on a sample of *n =* 648 because the questions on label credibility were only added after an initial data collection phase. All continuous variables were z‐standardized. Therefore, all β‐estimates are standardized and can be read as a measure of effect size. Posterior predictive plots were used to evaluate the model fit. There were no influential cases (Pareto k > .7). Rhat was <1.01, ESS > 1,000 for all relevant parameters and there were no divergent transitions. To compare the effect of sustainability labels on healthiness ratings with the effect of healthiness labels on sustainability a z‐test was performed using the cocor package (Diedenhofen & Musch, 2015). Note, that for the Bayesian analyses, no p‐values are reported. Instead, credible intervals provide the range of values in which the true estimate lies with a probability of 95% (Hespanhol et al., 2019). V‐Plots depicting the distributions were created using the v‐plot designer (Blumenschein et al., 2020). Data can be retrieved from psycharchives: 10.23668/psycharchives.16154.

## RESULTS

### Sample characteristics

Of the *N* = 712 participants in the final sample, *n* = 392 indicated to be female (55.1%), *n* = 317 indicated to be male (44.5%) and 3 indicated ‘other’ (0.4%). The mean age was *M* = 40.99 years (*SD* = 12.97, range = 18–84), with 20.9% between 18 and 29, 60.3% between 30 and 54, 18.1% between 55 and 64 and 0.7% older than 65 years. Concerning education, 14.6% did not complete upper secondary education, 51% completed upper secondary education and 34.4% completed post‐secondary education. The sample met the planned representativeness of the Austrian population in terms of education (OECD, 2023), but deviated slightly in terms of gender (Bundeskanzleramt Österreich, 2021) and age^1^ (Statistik Austria, 2022).

### Manipulation check

Foods with high healthiness scores were rated higher on healthiness (*M*_*high*_ = 6.59; *SD*_*high*_ = 1.62) compared to foods with low healthiness scores (*M*_*low*_ = 5.23; *SD*_*low*_ = 1.89; δ = 0.75; CI: [0.71; 0.79]). Foods with high sustainability scores were rated higher on sustainability (*M*_*high*_ = 6.69; *SD*_*high*_ = 1.64) compared to foods with low sustainability scores (*M*_*low*_ = 5.41; *SD*_*low*_ = 2.01; δ = 0.68; CI: [0.64; 0.72]). The manipulation of both the healthiness and the sustainability scores was therefore successful.

### Descriptive statistics

For an overview of descriptive statistics see Table 1.

**TABLE 1:** Means (M), standard deviations (SD) and correlations (*r*) among study variables (N = 712).

	*M (SD; scale range)*	*1* *(r)*	*2* *(r)*
Healthiness ratings	6.25 (1.7; 1–10)		
Sustainability ratings	5.97 (1.79; 1–10)		
Credibility of healthiness label	6.75 (2.22; 1–10)		
Credibility of sustainability label	6.56 (2.32; 1–10)		
1 general health interest	4.04 (1.18; 1–7)	‐	
2 general sustainability interest	3.08 (1.27; 1–7)	.67	‐
3 belief in relationship between healthiness and sustainability	3.66 (1.31; 1–7)	.26	.30

*Note*: For the credibility ratings n = 648.

### The effect of healthiness scores on sustainability ratings of food (H_1_)

**FIGURE 2:**
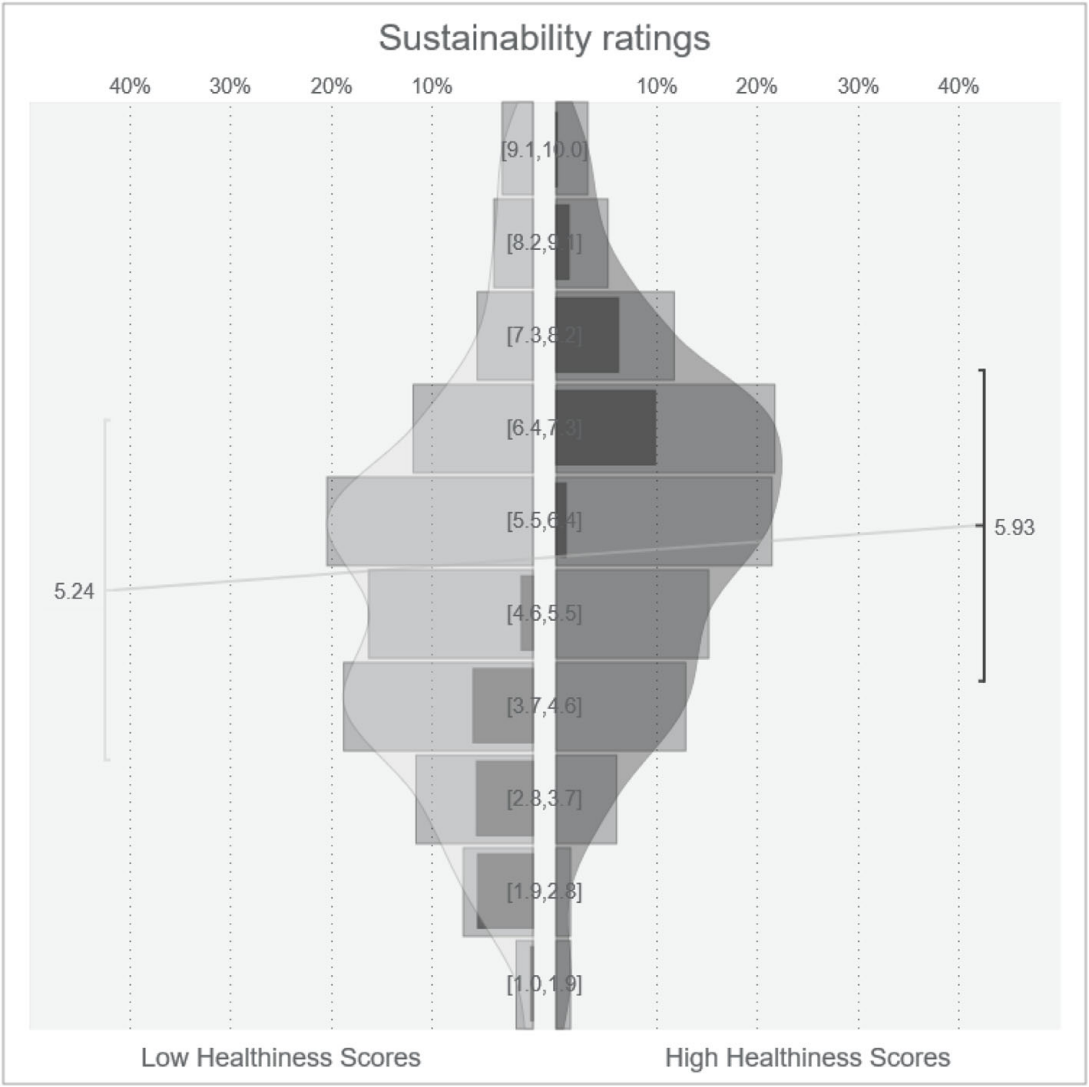
Sustainability ratings for foods with low and high healthiness scores. *Note.* V‐plot depicting the distribution, mean and standard deviation of sustainability for high and low healthiness scores.

The point‐biserial correlation between healthiness scores and sustainability ratings was *r* = .19. The Bayesian t‐test showed that sustainability ratings were higher when foods had high healthiness scores compared to low healthiness scores (*M*_*high*_ = 5.93; *M*_*low*_ = 5.24; δ = 0.39; CI: [0.36; 0.41]; for an overview of the distribution of sustainability ratings for high and low healthiness scores see figure 2). Bayesian linear models indicated that this effect was neither moderated by health interest (β = .03; CI: [−0.12; 0.18]), nor sustainability interest (β = −.03; CI: [−0.18; 0.11]), nor the strength of the belief in a ‘healthy = sustainable’ heuristic (β = 0.03; CI: [−0.11; 0.18]; see Table 2). Individuals with a higher belief in a relationship between healthiness and sustainability gave higher sustainability ratings, independent of the healthiness manipulation (β = .27; CI: [0.18; 0.37]). Taken together, H_1_ was confirmed, whereas there was no evidence for H_1a‐c._

**TABLE 2:** Effects of healthiness scores and moderating variables on sustainability ratings (N = 712/n = 648).

	Model without label credibility		Model with label credibility	
	Standardized estimate (Est.Error)	Credible interval	Standardized estimate (Est.Error)	Credible interval
Model including health interest as a moderator				
Intercept	**.20** (0.05)	**0.09–0.30**	**.22** (0.05)	**0.12 – 0.33**
Healthiness scores	**−.39** (0.07)	**−0.54 ‐to −0.24**	**−.43** (0.08)	−**0.58 – −0.28**
Health interest	.02 (0.05)	−0.08 – 0.12	.00 (0.05)	−0.11 – 0.11
Healthiness scores* health interest	.03 (0.07)	−0.12 – 0.18	−.06 (0.08)	−0.21 – 0.09
Health label credibility			**.34** (0.05)	**0.24 – 0.44**
Healthiness score * healthiness label credibility			−.01 (0.08)	−0.14 – 0.16
Health interest * healthiness label credibility			.02 (0.05)	−0.07 – 0.11
Healthiness score * health interest * healthiness label credibility			−.03 (0.07)	−0.17 – 0.12
Model including sustainability interest as a moderator				
Intercept	**.20** (0.05)	**0.09 – 0.30**	**.21** (0.05)	**0.10 – 0.32**
Healthiness scores	**−.39** (0.07)	**−0.53 – −0.24**	**−.43** (0.08)	**−0.58 – −0.28**
Sustainability interest	.05 (0.05)	−0.05 – 0.16	.02 (0.06)	−0.09 – 0.13
Healthiness scores* sustainability interest	−.03 (0.07)	−0.18 – 0.11	**−.14** (0.08)	**−0.29 – 0.01**
Healthiness label credibility			**.34** (0.05)	**0.23 – 0.45**
Healthiness score * healthiness label credibility			0.03 (0.08)	−0.13 – 0.18
Sustainability interest * healthiness label credibility			.08 (0.05)	−0.02 – 0.17
Healthiness score * sustainability interest * health label credibility			−.03 (0.07)	−0.16 – 0.10
Model including belief in relationship between healthiness and sustainability as a moderator				
Intercept	**.20** (0.05)	**0.10 − 0.30**	**.23** (0.05)	**0.13 – 0.3**
Healthiness scores	**−.39** (0.07)	**−0.54 – −0.25**	**−.50** (0.07)	**−0.65 – –0.36**
Belief in H‐S‐relationship	**.27** (0.05)	**0.18 – 0.37**	**.24** (0.05)	**0.13 – 0.34**
Healthiness scores* belief in H‐S‐relationship	.03 (0.07)	−0.11 – 0.18	−.03 (0.07)	−0.18 – 0.11
Healthiness label credibility			**.29** (0.05)	**0.19 – 0.40**
Healthiness score * Healthiness label credibility			.01 (0.08)	−0.14 – 0.16
Belief in H‐S‐relationship * healthiness label credibility			.03 (0.04)	−0.05 – 0.11
Healthiness score * belief in H‐S‐relationship* Healthiness label credibility			**.15 (0.06)**	**0.03 – 0.27**

*Note*: Results of the Bayesian linear models for the moderating effects of health interest, sustainability interest and strength of the belief in a relationship between healthiness and sustainability on the relationship between healthiness scores and sustainability ratings. Bold numbers indicate statistical significance. Belief in H‐S‐relationship = Belief in a relationship between healthiness and sustainability.

**FIGURE 3:**
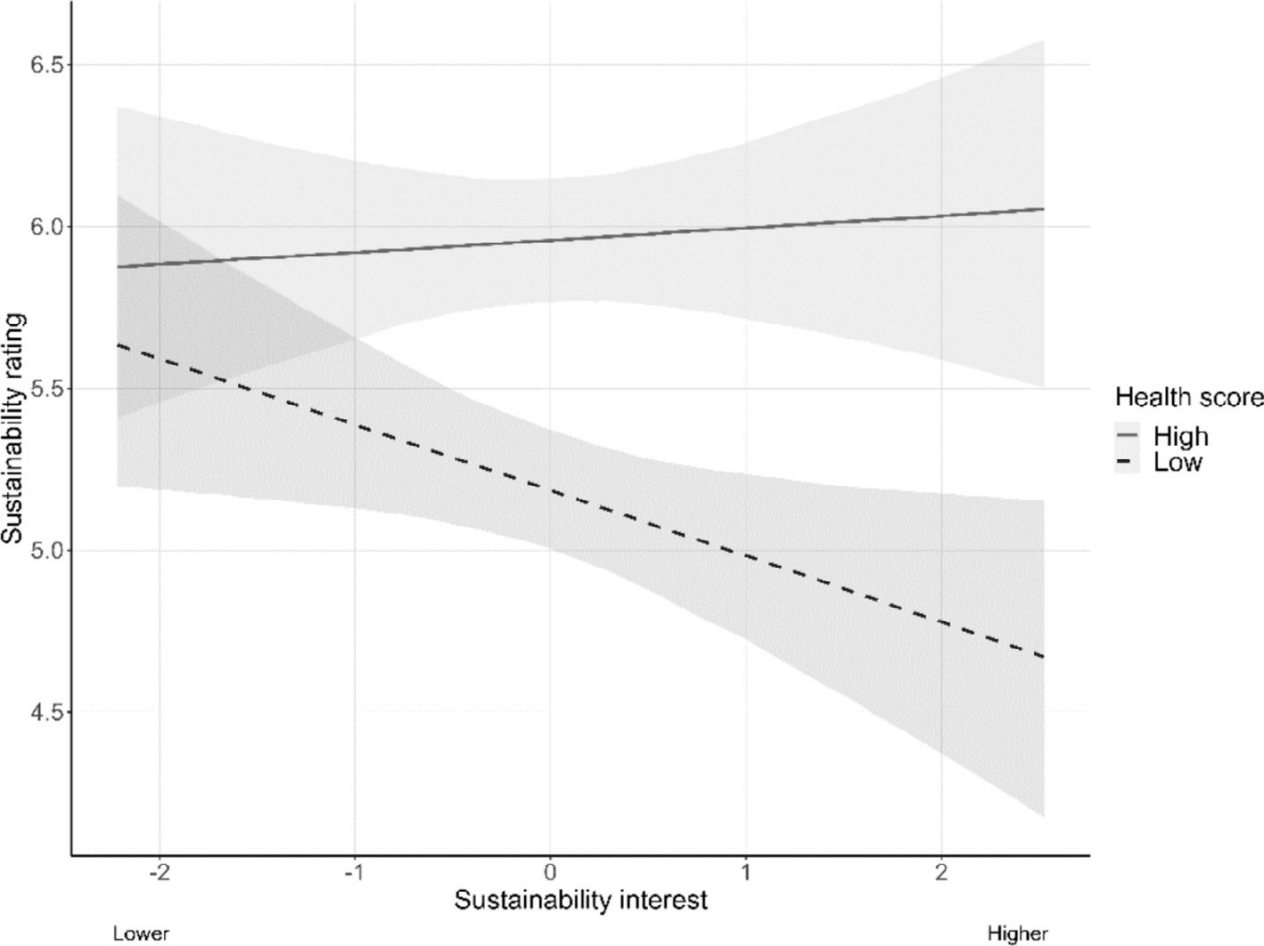
Effect of health scores on sustainability ratings moderated by sustainability interest (controlled for credibility). *Note.* Results of the Bayesian linear model for the effects of healthiness scores and sustainability interest on sustainability ratings controlled for label credibility. Sustainability interest was z‐standardized. Sustainability ratings were z‐standardized in the analysis but are plotted in the original scale for illustrative purposes. Ratings were on a scale from 1 (not at all sustainable) to 10 (totally sustainable). Error bands display credible intervals.

The additional exploratory analyses including label credibility confirmed the effect of healthiness scores on sustainability ratings for both individuals with higher credibility ratings (≥6; *n = 482; M*_*high*_ = 6.31; *M*_*low*_ = 5.47; δ = 0.49; CI: [0.46; 0.53]) and individuals with lower credibility ratings (≤5; *n* = 166; *M*_*high*_ = 4.99; *M*_*low*_ = 4.49; δ = 0.27; CI: [0.19; 0.33]). However, results for the moderating variables changed in part. The effect of healthiness scores on sustainability ratings was larger in individuals with a higher interest in sustainability (healthiness score x sustainability interest; β = −.14; CI: [−0.29; 0.01]; note that the credibility interval includes 0 and therefore includes a small possibility of the estimate being 0, controlling for credibility (Figure 3). The moderating analysis of the belief in a relationship between healthiness and sustainability showed no moderation when label credibility was higher, but a stronger effect of health scores in individuals with a stronger belief in the relationship and lower label credibility (health score x belief in relationship x label credibility; β = .15; CI: [0.03; 0.27]). In sum, taking label credibility into account there was evidence for H_1_ and H_1b_.

### The effect of sustainability scores on healthiness ratings of food (H_2_)

**FIGURE 4:**
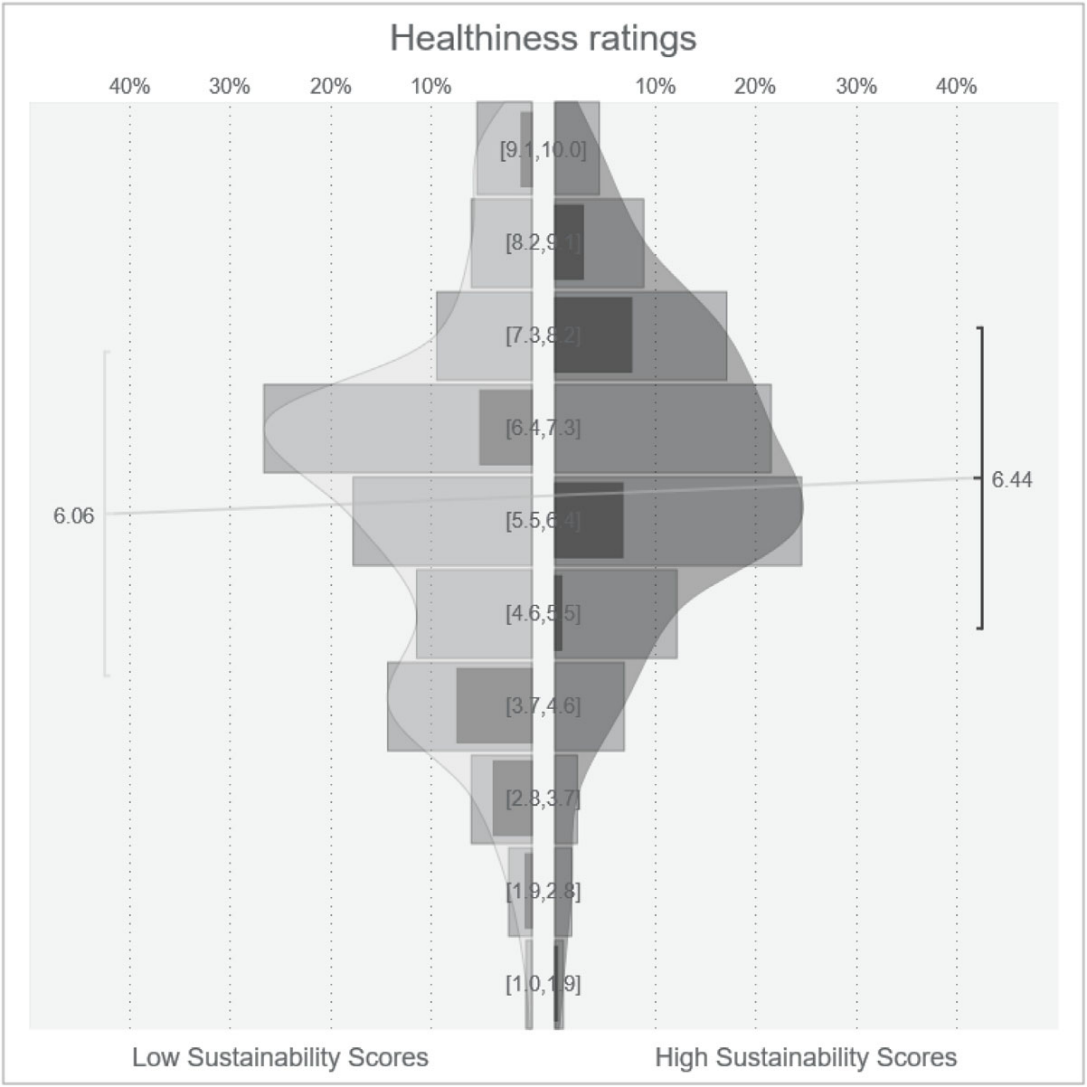
Healthiness ratings for foods with low and high sustainability scores. *Note.* V‐plot depicting the distribution, mean and standard deviation of healthiness for high and low sustainability scores.

The point‐biserial correlation between sustainability scores and healthiness ratings was *r* = .11. The Bayesian t‐test showed higher healthiness ratings for foods labelled with high sustainability scores compared to low sustainability scores (*M*_*high*_ = 6.44; *M*_*low*_ = 6.06; δ = 0.22; CI: [0.2; 0.24]; for an overview of the distribution of healthiness ratings for high and low sustainability scores see Figure 4). The effect was increased by interest in sustainability (β = −.14; CI: [−0.28; 0.01], see Figure 2 and Table 3): individuals with a higher interest in sustainability rated the healthiness of foods with high sustainability score higher. Neither interest in health (β = −.10; CI: [−0.25; 0.05]) nor the strength of belief in a relationship between healthiness and sustainability (β = −.05; CI: [−0.19; 0.09]; see Table 3) moderated the relationship. Individuals with a higher belief in a relationship between healthiness and sustainability gave higher health ratings, independent of the sustainability manipulation (β = .27; CI: [0.17; 0.37]). In sum, H_2_ and H_2b_ were confirmed, whereas there was no evidence for H_2a_ and H_2c._ The additional exploratory analyses including label credibility confirmed the effect of sustainability scores on healthiness ratings for both individuals with higher credibility ratings (≥6; *n* = 435; *M*_*high*_ = 6.77; *M*_*low*_ = 6.32; δ = 0.27; CI: [0.24; 0.29]) and individuals with lower credibility ratings (≤5; *n* = 213; *M*_*high*_ = 5.82; *M*_*low*_ = 5.41; δ = 0.25; CI: [0.20; 0.30]). However, the moderating effect of sustainability interest became non‐significant when including label credibility (sustainability score x sustainability interest; β = −.07; CI: [−0.22; 0.08]).

**TABLE 3:** Effects of sustainability scores and moderating variables on healthiness ratings (N = 712/n = 648).

	Model without label credibility		Model with label credibility	
	Standardized estimate (Est. Error)	Credible interval	Standardized estimate (Est. Error)	Credible interval
Model including health interest as a moderator				
Intercept	**.11** (0.05)	**0.01– 0.21**	**.12** (0.05)	**0.01 – ‐0.22**
Sustainability scores	**−.23** (0.07)	−**0.37 ‐ −0.08**	**−.24** (0.07)	**−0.38 ‐ −0.10**
Healthiness interest	−.03 (0.05)	−0.13 – 0.07	−.**14** (0.05)	**−0.25 ‐ −0.04**
Sustainability scores * health interest	−.10 (0.08)	−0.25 – 0.05	−.04 (0.08)	−0.19 – 0.11
Sustainability label credibility			**.42** (0.05)	**0.32 – 0.52**
Sustainability scores * sustainability label credibility			−.07 (0.07)	−0.22 – 0.08
Health interest *sustainability label credibility			−.02 (0.04)	−0.11 – 0.07
Sustainability scores * health interest *sustainability label credibility			−.04 (0.07)	−0.18 – 0.09
Model including sustainability interest as a moderator				
Intercept	**.11** (0.05)	**0.01– 0.22**	**.12** (0.05)	**0.01 – ‐0.22**
Sustainability scores	**−.23** (0.08)	**−0.37 ‐ −0.08**	**−.22** (0.08)	**−0.37 ‐ −0.07**
Sustainability interest	.05 (0.05)	−0.05 – 0.15	**−11** (0.05)	**−0.22 ‐ −0.00**
Sustainability scores * sustainability interest	**−.14** (0.07)	**−0.28 – 0.01**	−.07 (0.08)	‐0.22 – 0.08
Sustainability label credibility			**.42** (0.05)	**0.32 – 0.52**
Sustainability scores *sustainability label credibility			−.06 (0.08)	−0.21 – 0.09
Sustainability interest *sustainability label credibility			−.01 (0.04)	− 0.09 – 0.07
Sustainability scores * sustainability interest *sustainability label credibility			−.08 (0.06)	−0.20 – 0.05
Model including belief in the relationship between healthiness and sustainability as a moderator				
Intercept	**.10** (0.05)	**0.00 – 0.20**	**.12** (0.05)	**0.01 – 0.23**
Sustainability scores	**−.21** (0.07)	**−0.35 ‐ −0.08**	**−.26** (0.07)	**−0.41 ‐ ‐0.11**
Belief in H‐S‐relationship	**.27** (0.05)	**0.17 – 0.37**	**.15** (0.05)	**0.04 – 0.25**
Sustainability scores * belief in H‐S‐relationship	−.05 (0.07)	−0.19 – 0.09	−.02 (0.08)	−0.16 – 0.13
Sustainability label credibility			.**33** (0.05)	**0.23 – 0.43**
Sustainability scores *sustainability label credibility			−.04 (0.08)	−0.19 – 0.11
Belief in H‐S‐relationship *sustainability label credibility			−.03 (0.04)	−0.12 – 0.05
Sustainability scores * sustainability interest *sustainability label credibility			−.09 (0.07)	−0.04 – 0.22

*Note*: Results of the Bayesian linear models for the moderating effects of health interest, sustainability interest and strength of the belief in a relationship between healthiness and sustainability on the relationship between sustainability scores and healthiness ratings. Bold numbers indicate statistical significance. Belief in H‐S‐relationship = Belief in a relationship between healthiness and sustainability.

### The difference between the effect of healthiness scores on sustainability ratings and the effect of sustainability scores on healthiness ratings (H_3_)

The z‐test testing whether there is a difference between the effect of healthiness scores on sustainability ratings (*r* = .19) and the effect of sustainability scores on healthiness ratings (*r* = .11) was marginally significant (z = 1.55, *p* = .06). H_3_ was therefore only marginally confirmed. Results remained the same for individuals with higher average label credibility (≥ 6: *z =* 1.64; *p* = .05) and were non‐significant for individuals with lower average label credibility (≤5; *z* = 0.86; *p =* .19).

## DISCUSSION

The present study experimentally manipulated healthiness and sustainability scores to investigate the presence and direction of the previously observed ‘healthy = sustainable’ heuristic. Individual differences considering the interest in health and sustainability and the belief in a relationship between the healthiness and sustainability of foods were taken into account.

### Evidence for the ‘healthy = sustainable’ heuristic

While previous studies observed a relationship between the perceived healthiness and sustainability of foods (see e.g., Lazzarini et al., 2016; Sproesser, Arens‐Azevedo, & Renner, 2023), it was not clear whether the underlying mechanism for this was indeed a ‘healthy = sustainable’ heuristic or whether a third variable was responsible for the observed correlations between healthiness and sustainability ratings. Our study was able to show that experimentally manipulating the perceived healthiness of foods changed the perceived sustainability and vice versa. This indicates that individuals indeed make use of heuristic processing, linking the two dimensions. While our results showed that the heuristic works bi‐directionally, it appears that the influence of healthiness on sustainability ratings is marginally larger than the effect of sustainability on healthiness. There are multiple potential reasons for this.

One possibility, as Sproesser, Aulbach, et al. (2023) argued, is that people are more familiar with the concept of healthy foods compared to sustainable foods and are therefore more likely to use indicators of healthiness as a cue for sustainability ratings than vice versa. In favour of this view are the results of a study by Wassmann et al. (2023). When asked to assemble a sustainable meal, participants selected fewer grams from desserts compared to the control group, pointing towards the use of a ‘healthy = sustainable’ heuristic. The same strategy (fewer desserts) was also applied when individuals were asked to assemble healthy meals (Bucher et al., 2011). In addition, whereas previous research indicates that consumers are knowledgeable about the general concept of healthy eating (see e.g., Moraes et al., 2023), they do not have a good understanding of the concept of sustainability (Grunert et al., 2014). These factors may have contributed to our results of a smaller effect of sustainability labels on healthiness ratings.

Another possibility is that the influence of healthiness labels was larger because individuals in our sample were somewhat more interested in health than they were in sustainability (*M*_*Health*_ = 4.04; *M*_*Sus*_ = 3.08; on a scale from 1 to 7). Individuals might therefore have paid more attention and given more importance to healthiness labels than to sustainability labels. This would fit to our finding that the influence of sustainability labels on health ratings was larger in those with a higher sustainability interest. Previous research showed that low health interest was associated with lower visual attention to health labels (Fenko et al., 2018). This also may have been the case for our sustainability labels. Future studies need to investigate whether our results change in samples with a higher interest in sustainability. Also, it might be interesting to have a measure of attendance to the labels, for example using eye‐tracking.

### Moderating effects of interests and beliefs on the ‘healthy = sustainable’ heuristic

Our initial analysis revealed that neither health nor sustainability interest moderated the effect of health scores on sustainability ratings. However, when controlling for label credibility, interest in sustainability did play a role in individuals who found the healthiness labels more credible. For the effect of sustainability scores on health ratings the opposite pattern emerged: while the initial analysis showed a stronger effect of sustainability scores in individuals with a higher sustainability interest, this disappeared when controlling for credibility. In addition to the inconclusive findings in previous studies, this suggests that the moderating effects of interests (both related to the halo‐producing attribute and the outcome) are possibly rather small and of limited practical implications. Future studies might profit from investigating other possible moderators such as actual knowledge about the healthiness and sustainability of foods (cf. Sproesser, Aulbach, et al., 2023). Given that heuristic processes are mainly used under uncertainty (Tversky & Kahneman, 1974), knowledge might reduce them and be more influential than interest alone.

Interestingly, the explicit belief in a relationship between healthiness and sustainability of foods did not moderate the relationships between label scores and ratings. It would seem plausible that, for individuals who hold such a belief, there is a stronger effect than for those who do not hold such a belief. This was also found in related studies investigating the ‘unhealthy = tasty’ relationship (Haasova & Florack, 2019; Raghunathan et al., 2006). In contrast, the results of this study did not only show an effect of label scores on ratings although the overall explicit belief in a relationship was relatively low (3.7/7), they also showed that the effect was not stronger in individuals with a stronger belief in a relationship. It is conceivable that the ‘healthy = sustainable’ heuristic operates on a more implicit level as compared to explicit cognitions. This would also be in line with the findings of Wassmann et al. (2023). They found participants to choose fewer desserts when assembling a sustainable compared to a control meal, which can be seen as a strategy to increase healthiness (Bucher et al., 2011). Yet, when asked why they chose the foods, healthiness was not mentioned more often than for the control meal. This might indicate that individuals were not aware of using healthiness as a cue for sustainable choices. A future study might investigate the heuristic on an implicit level, e.g., using the implicit association test (Greenwald et al., 1998). Another possibility for the absence of a moderating effect of the belief in a relationship between healthiness and sustainability is that our three‐item scale did not succeed in adequately measuring the concept.

### Label credibility

Participants rated labels as credible on average (*M*_*Health*_ = 6.75; *SD*_*Health*_ = 2.22; *Mdn*_*Health*_ = 7 *M*_*Sus*_ = 6.56; *SD*_*Health*_ = 2.32; *Mdn*_*Sus*_ = 7; on a scale from 1 to 10). While this shows that most participants believed in the label scores, there were still around 25% of participants rating label credibility as ≤ 5/10. We had preregistered neither excluding participants who gave low credibility scores nor statistically controlling for label credibility; therefore, all analyses accounting for credibility were exploratory. Importantly, this did not change the main results of label effects. It did however change the moderating role of interests in some instances. Unfortunately, there were no comments hinting at why people gave lower credibility ratings in our study's open comment section. A reason might be that consumers tend to be more sceptical about labels they are unfamiliar with and labels with more general claims (Sirieix et al., 2013), which applies to our study. The results imply that label credibility should be measured and controlled for in future studies and research about how to improve the credibility of healthiness and sustainability labels is needed. In addition, it would be of interest to investigate whether individuals with low label credibility do not show halo effects as a consequence or whether they are still affected by it. Given that our main effects of label score remained significant in the group with low label credibility (≤5), there is some indication that some degree of label effect is retained in that group.

### Implications

The study confirmed the existence of a ‘healthy = sustainable’ heuristic and previous studies showing that labels can influence judgements of attributes beyond their purpose. This has clear relevance to everyday life when foods labelled with some type of sustainability indicator (e.g., ‘regionally produced’ or ‘organic’) are taken to be healthy or vice versa foods with a type of health label (e.g., the nutri‐score) are taken to be ‘good for the environment’. To avoid these label‐induced halo effects dual labelling might be beneficial. In the studies' open comment section many participants mentioned they would welcome sustainability labelling on foods. Using dual labels (health and sustainability) would be a straightforward implication of our results but requires further research. On the one hand, Sonntag et al. (2023) found consumers to deal well with two different label types on the same product, even if their information was conflicting (i.e., one label informed about a positive aspect, the other about a negative aspect). On the other hand, de Bauw et al. (2021) found that using a nutri‐score and an eco‐score in parallel leads to healthier choices but not more sustainable choices. Further research is needed to find an effective way to communicate both dimensions to consumers and could help to promote both personal as well as planetary health in line with the United Nations Sustainable Development Goals (i.e, Good Health and Wellbeing; Climate Action; United Nations, 2015). Moreover, an alternative to dual labelling might be to increase the knowledge about healthy but especially sustainable eating. Lazzarini et al. (2018) found that giving individuals a short list of guidelines about sustainable foods was more beneficial for sustainable choices than a sustainability label. Still, more studies about the effect of knowledge on the ‘healthy = sustainable’ heuristic and food choice are required.

### Limitations and outlook

The first limitation of the present study is the mean credibility of the labels being only 6.5/10 and 6.75/10 (*Mdn =* 7 for both). While this can still be considered as overall credible and we controlled the analysis for label credibility, future studies could try to further improve label credibility. Possibly this can be achieved by using a design that participants are already familiar with, such as the Nutri‐score in many EU member states (see e.g., Sirieix et al., 2013). Although the label design in our study had been successfully used previously (Cho & Baskin, 2018), some participants mentioned they found the design not very appealing. This again might be solved with a more colourful traffic‐light design. Second, the study was conducted in Austria and it is possible that there exist cross‐cultural differences (Sproesser et al., 2022; Werle et al., 2013). Third, our study used different types of food groups (breakfast, lunch/dinner, snacks). While this is beneficial in terms of generalization of our findings, the size of the stimulus set does not allow to draw conclusions about possible differences in the halo effect for different food groups. Fourth, to make the manipulation more credible, we used foods that were rated as ‘medium’ on both healthiness and sustainability in our pre‐test. Future studies might investigate if label effects persist for foods that differ on these dimensions, e.g., that are healthy but unsustainable. Fifth, the sample of the pre‐test differed from the sample of the main study in that it mainly consisted of mostly female university students who probably had higher food knowledge. However, we do not expect this to impact stimuli credibility for participants with less knowledge. Last, we did not use images of packaged foods, which might be the food group for which consumers are most used to labels, which could have affected results. Hence, future research needs to examine whether the present results differ as a function of food groups.

### Conclusions

The study expanded previous correlational results (Lazzarini et al., 2016; Sproesser, Arens‐Azevedo, & Renner, 2023) regarding the presence of a ‘healthy = sustainable’ heuristic with experimental evidence of a causal relationship between the judgements of the two attributes in a large, relatively representative sample. Healthiness perceptions tend to have a stronger influence on sustainability than vice versa. To avoid unjustified halo effects in food choice, more research on effective double labelling (i.e., healthiness and sustainability) as well as about the effect of knowledge on the ‘healthy = sustainable’ heuristic is needed.

## CONFLICT OF INTEREST STATEMENT

The authors have no conflicts to declare.

## ETHICS STATEMENT

The study was approved by the Ethics Committee of Johannes Kepler University Linz.

## Supporting information


**Table S1** The effect of health score and food type on sustainability ratings.
**Table S2** Bonferroni‐corrected post hoc tests for the effect of health score and food type on sustainability ratings.
**Table S3** The effect of sustainability score and food type on healthiness ratings.

## Data Availability

Data can be retrieved from psycharchives: 10.23668/psycharchives.16154

## Footnotes

^1^
Sample characteristics differ from a representative Austrian sample in the following dimensions (percentage sample / percentage population): female gender: 55.1/50.8%; age 18–29: 20.9/17.5%; age 40–54: 60.3/42%; age 55–64: 18.1/17.5%; age ≥65: 0.7/23%.
