# Supplementary material for: The ‘healthy = sustainable’ heuristic: Effects of health and sustainability labels on perceived sustainability and healthiness of foods
Source: Appl Psychol Health Well Being. 2025 May 7;17(3):e70031. doi: 10.1111/aphw.70031 (PMC12120393; doi:10.1111/aphw.70031)
Supplement: Supplementary file 1 — Table S1 The effect of health score and food type on sustainability ratings. Table S2 Bonferroni‐corrected post hoc tests for the effect of health score and food type on sustainability ratings. Table S3 The effect of sustainability score and food type on healthiness ratings. [file APHW-17-0-s001.docx]

**Supplement**

**Images**

High healthiness score

**
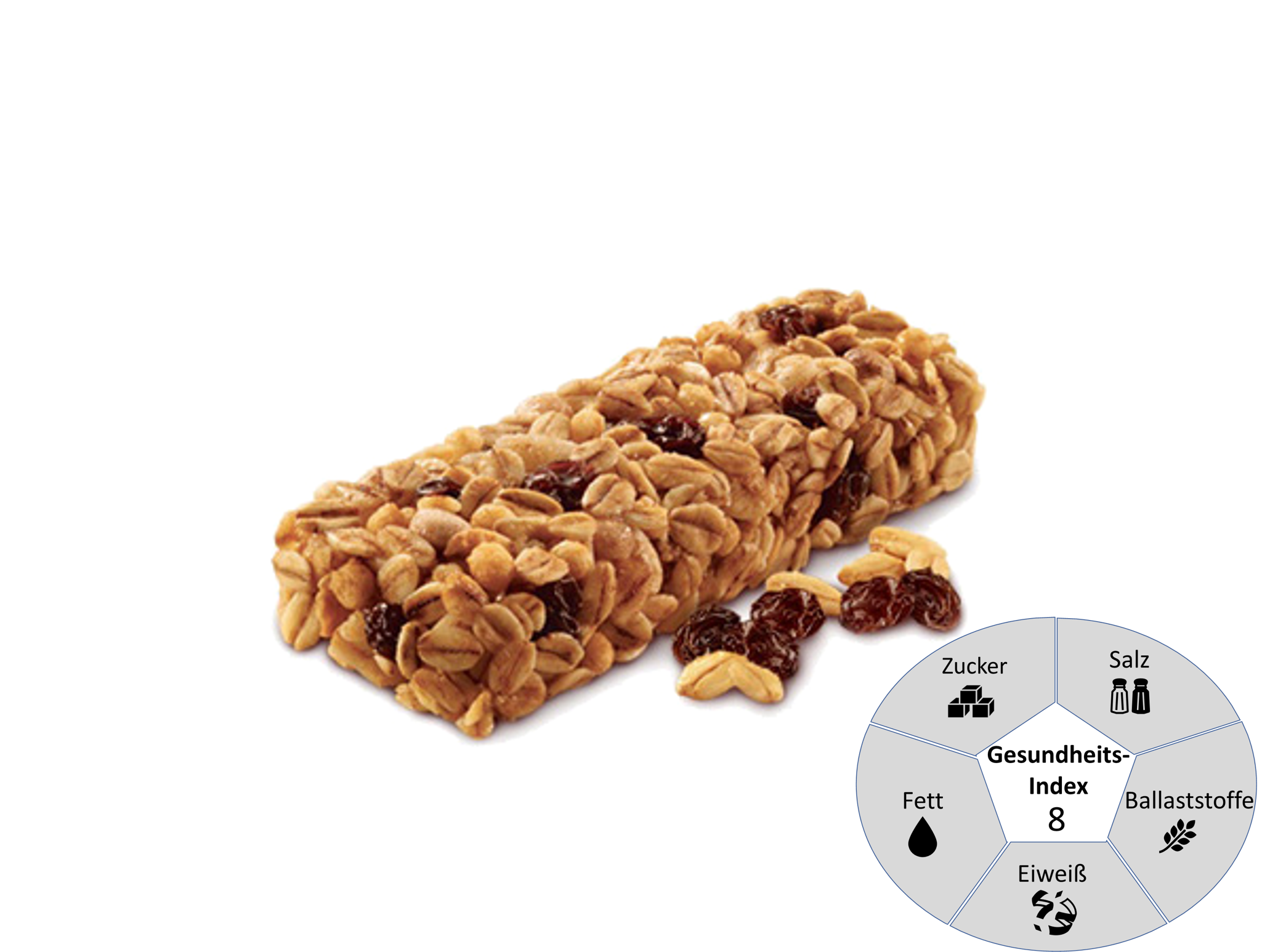

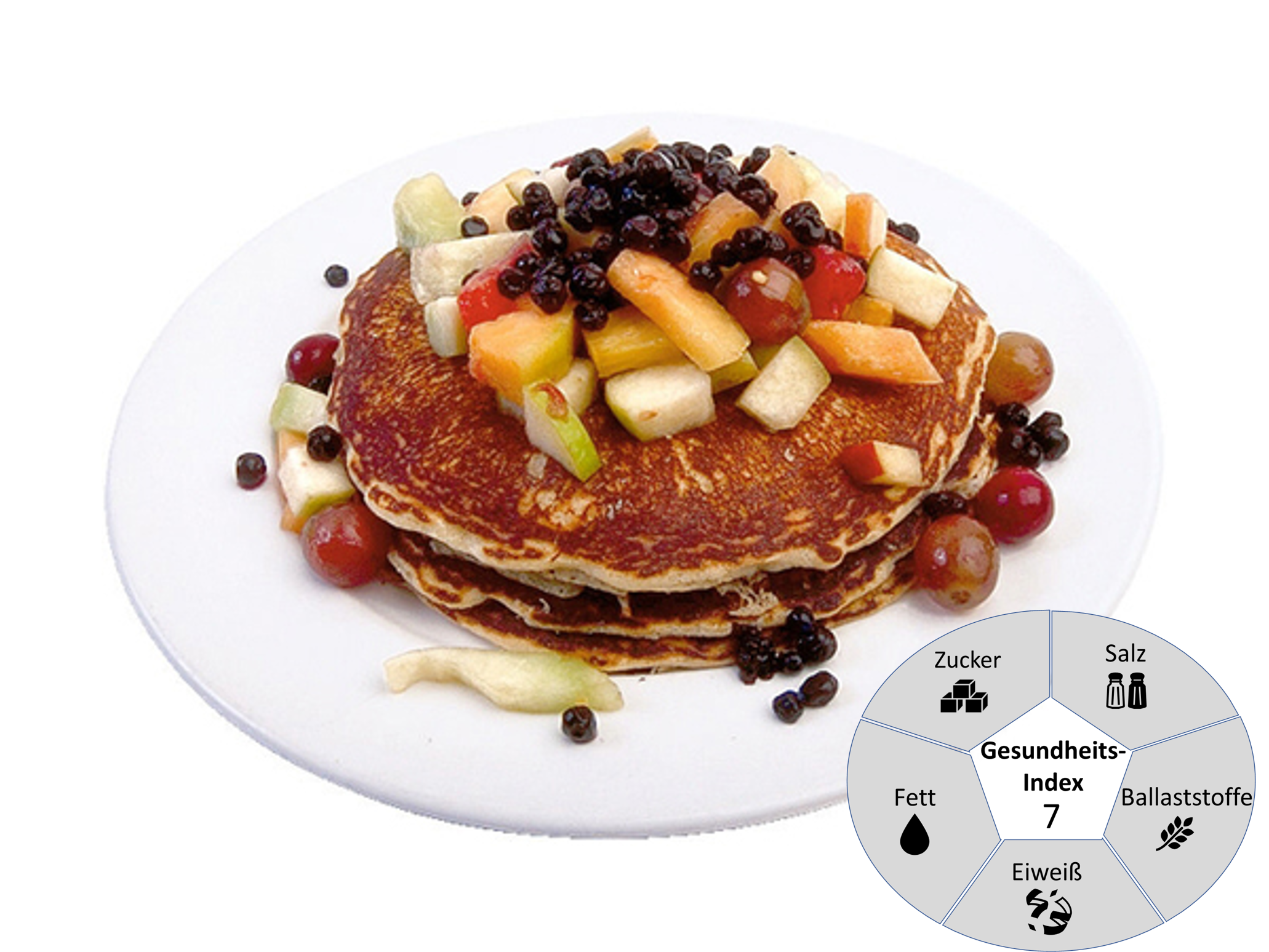

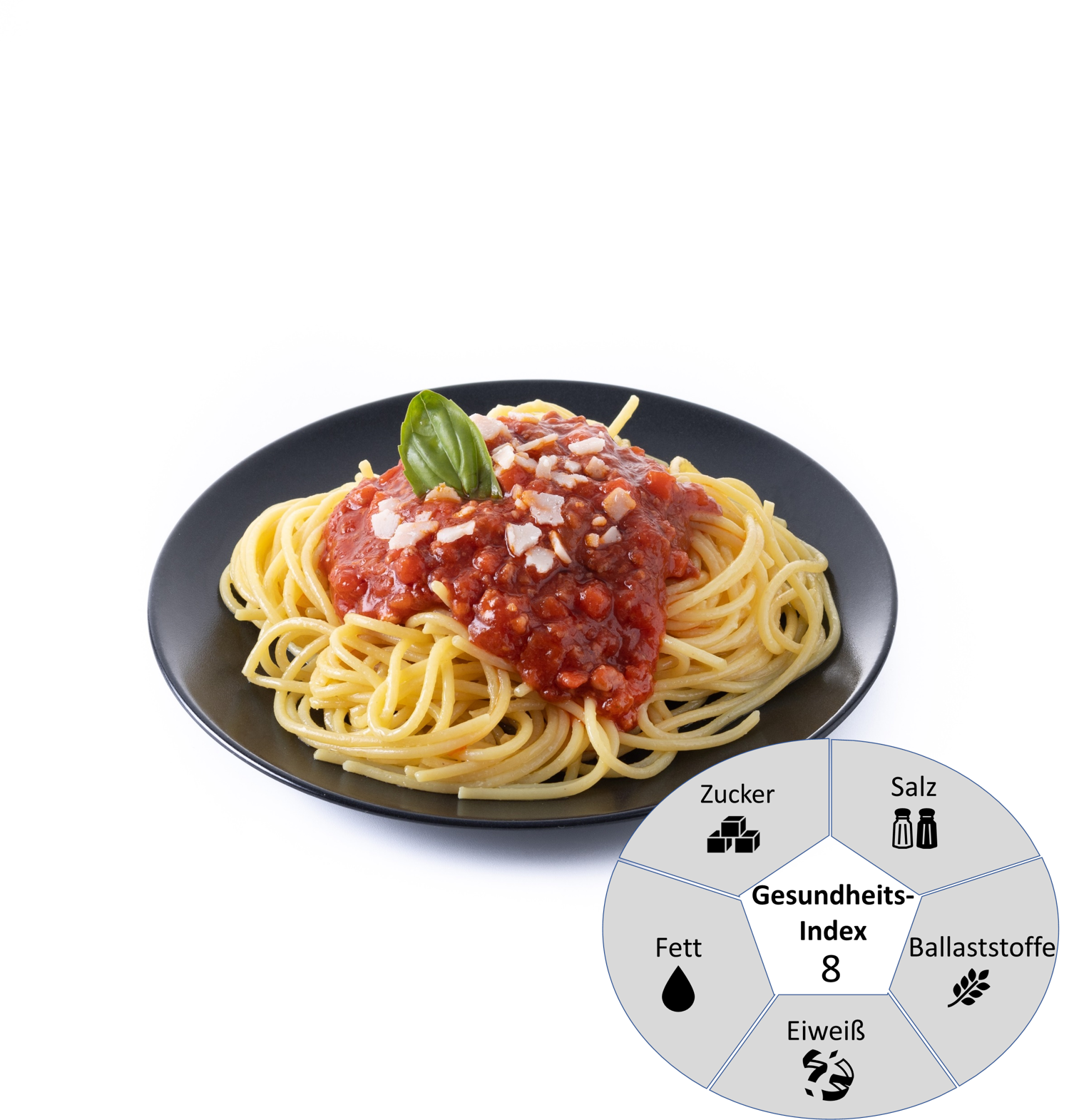

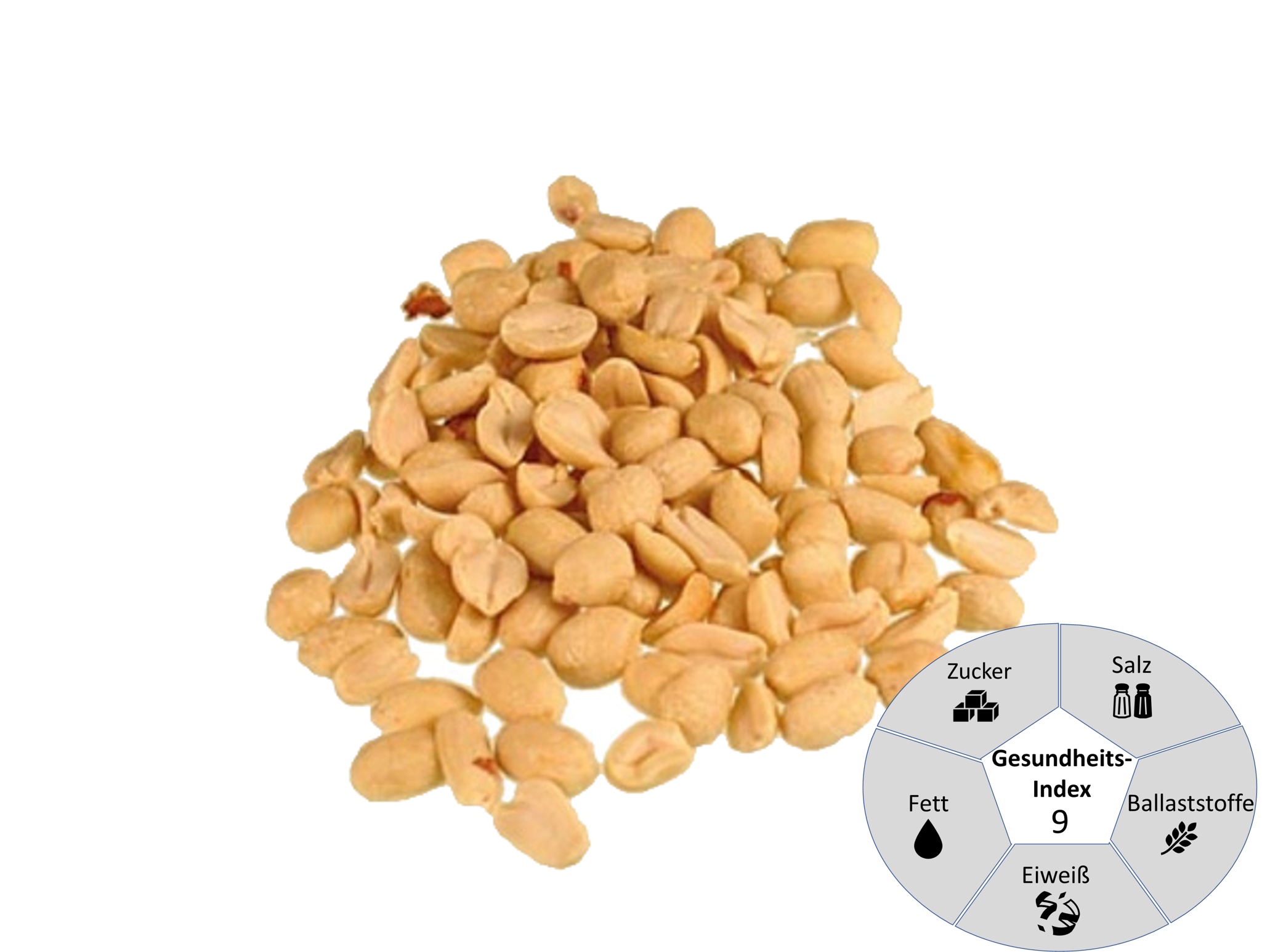
**

Low healthiness score


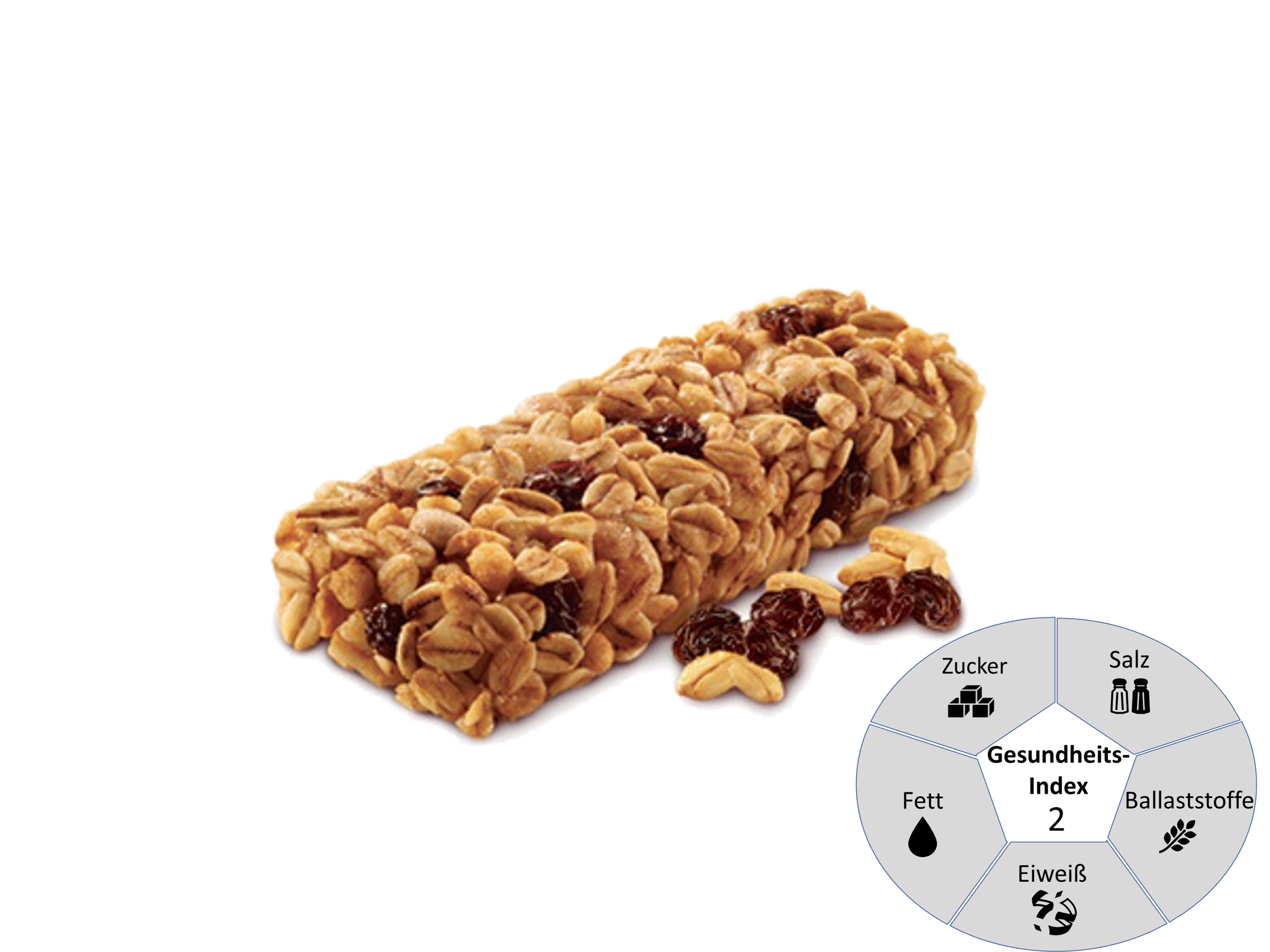

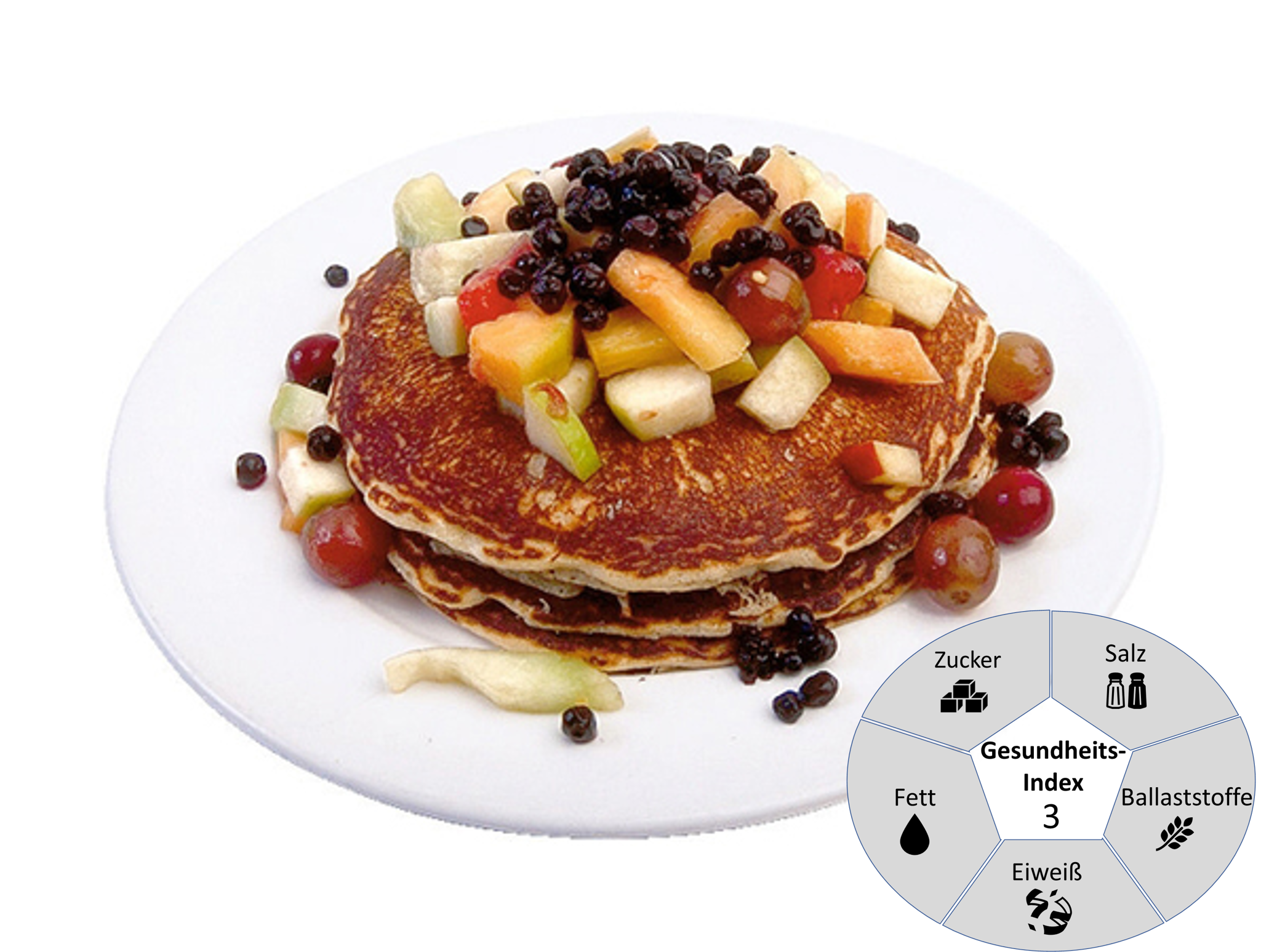

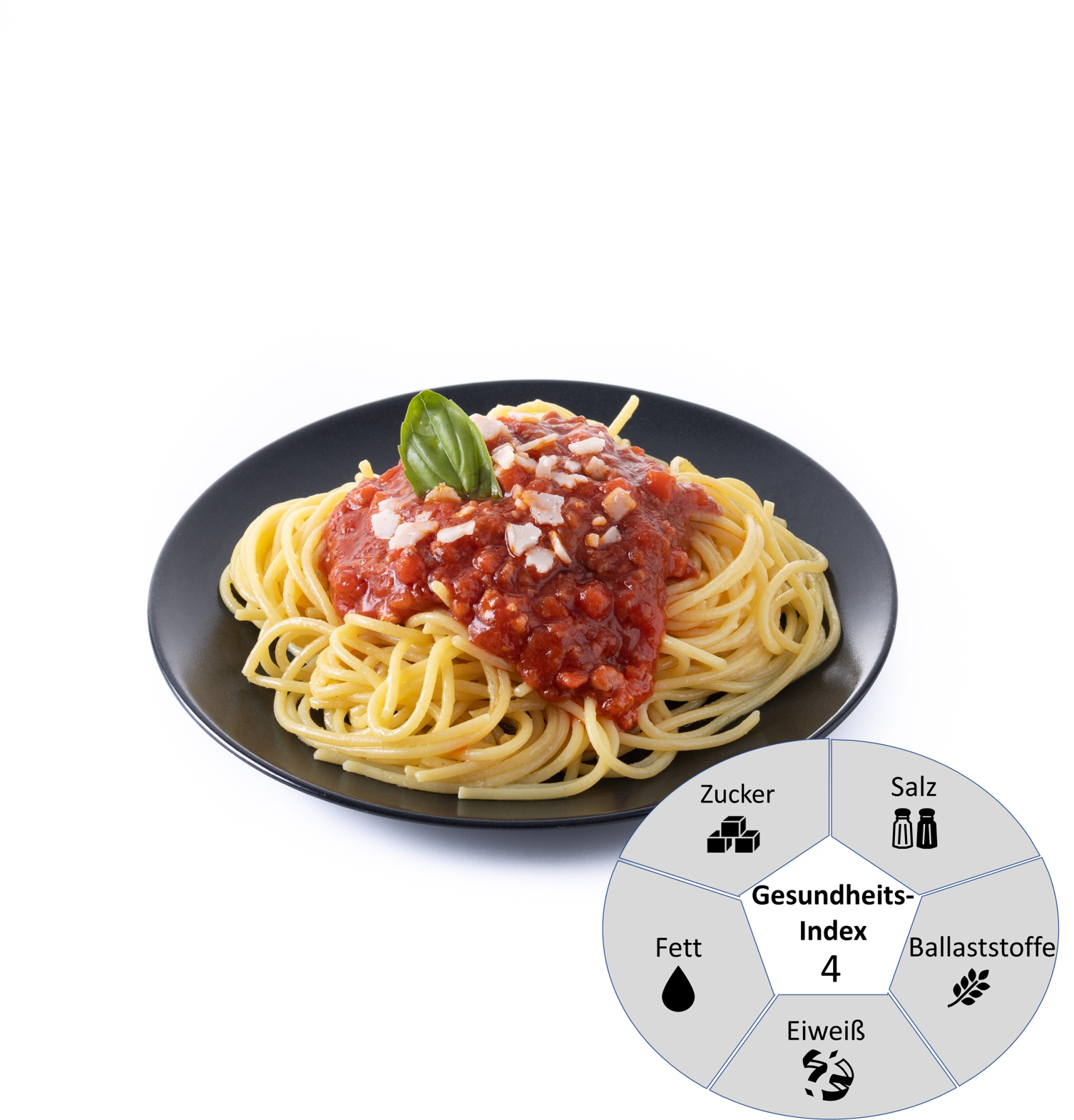

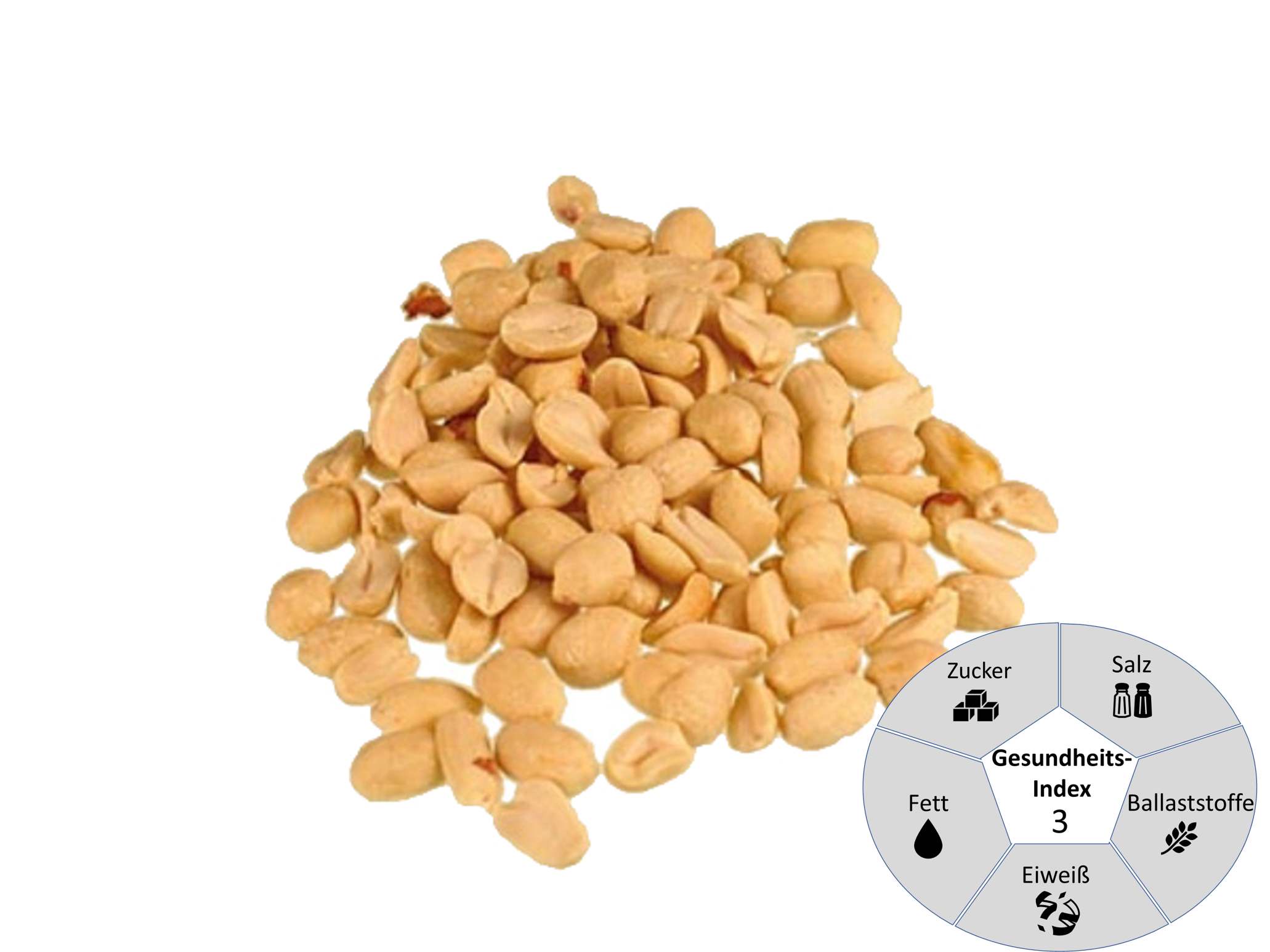


High sustainability score


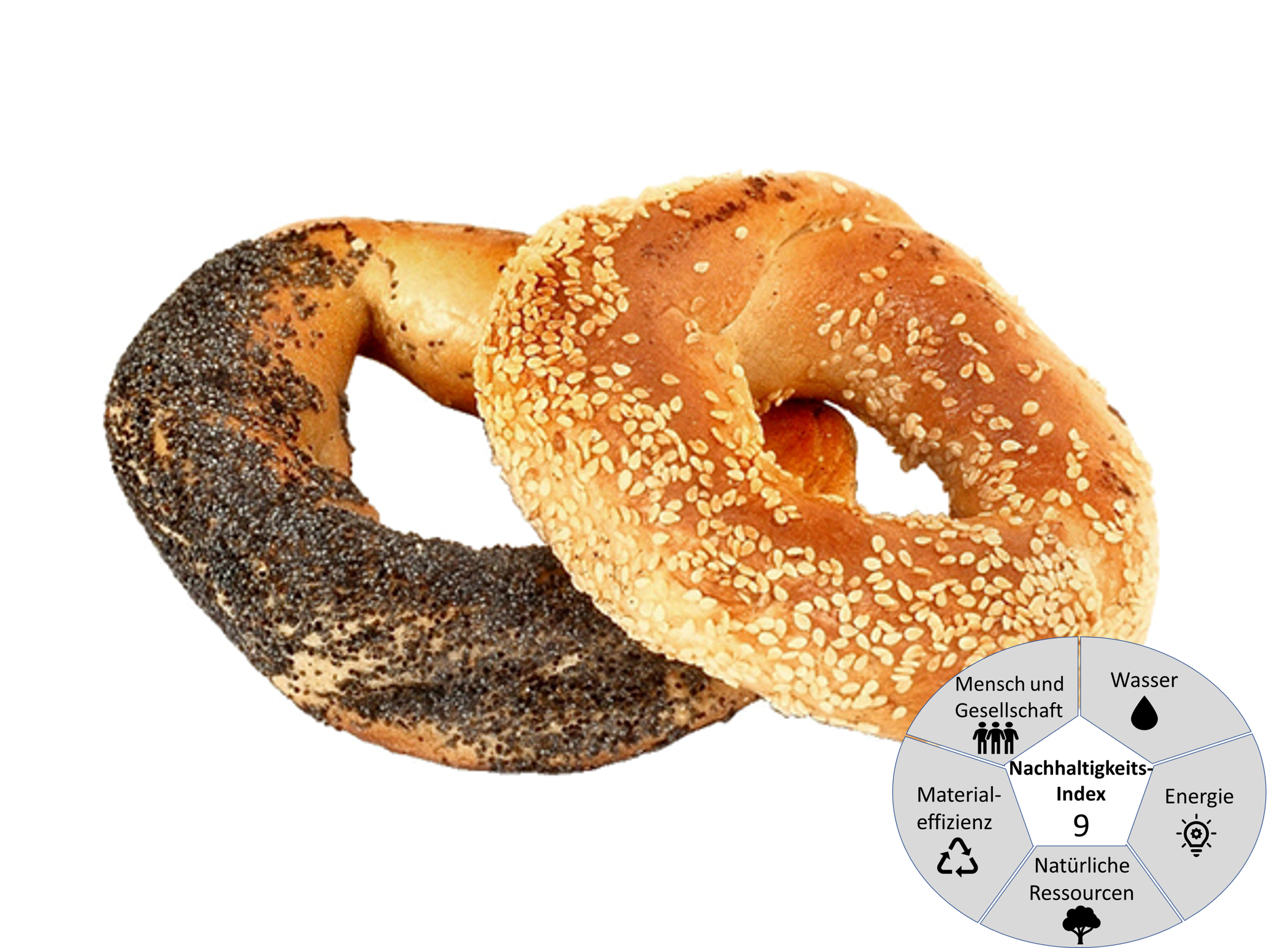

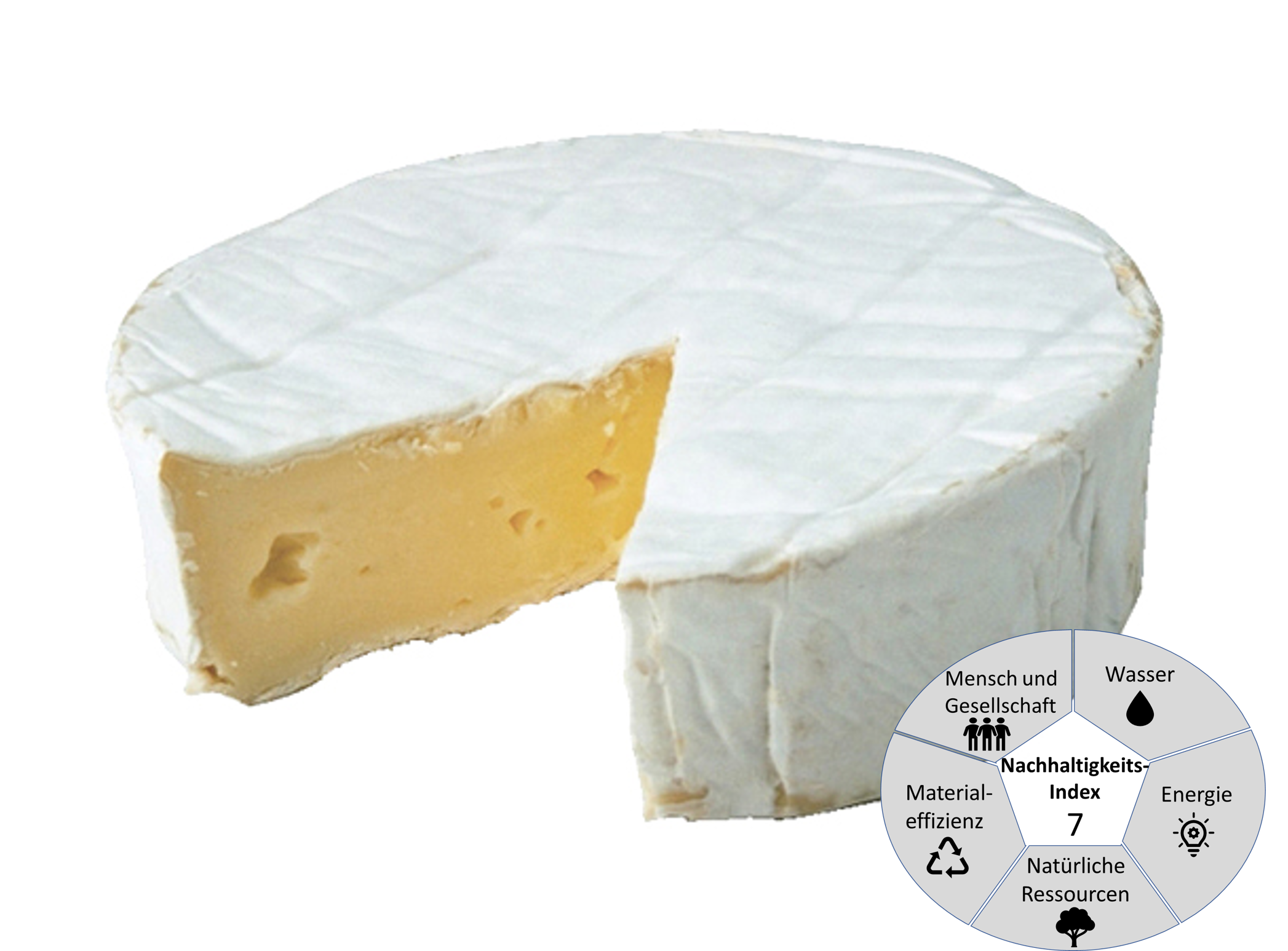

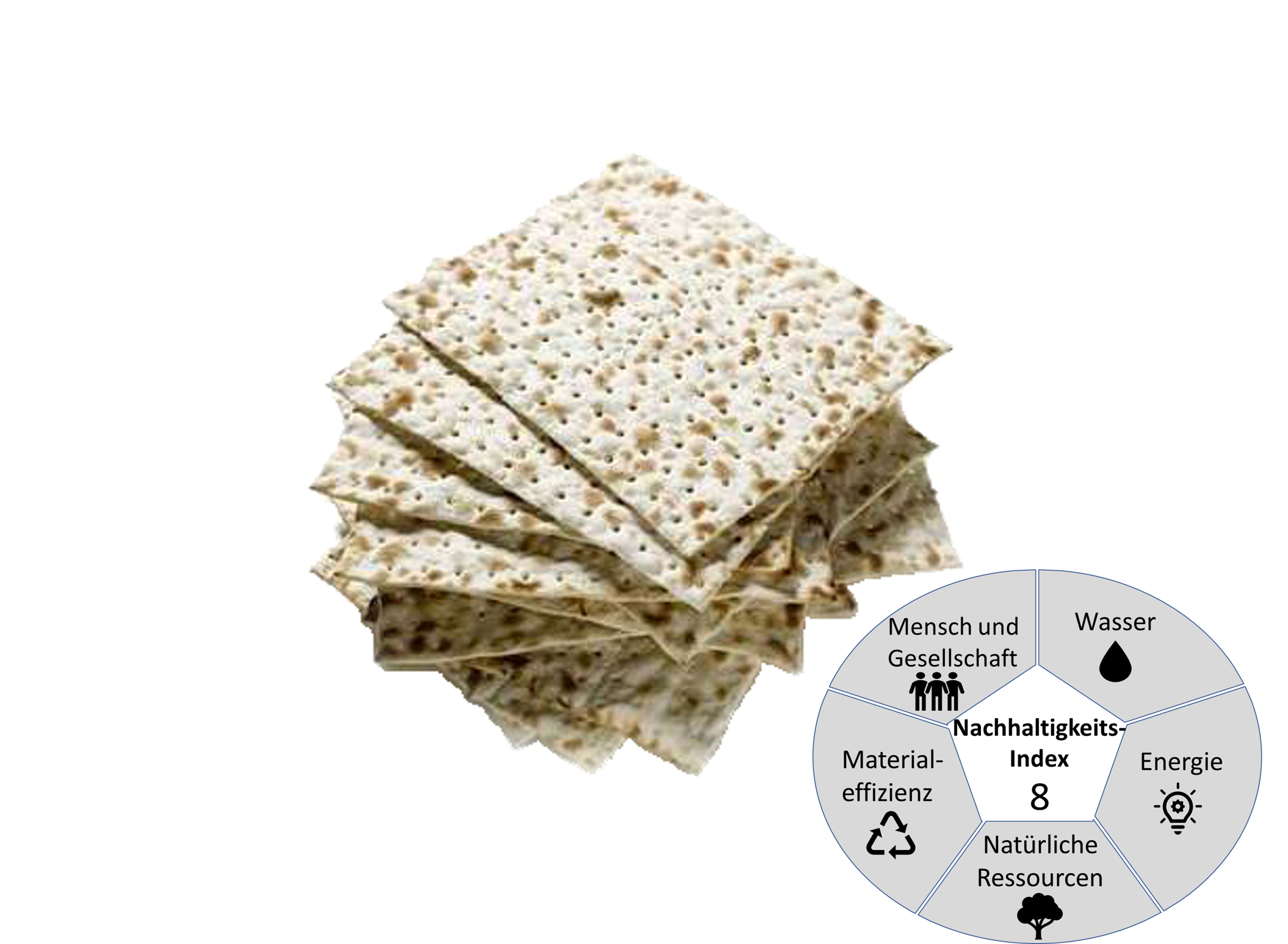

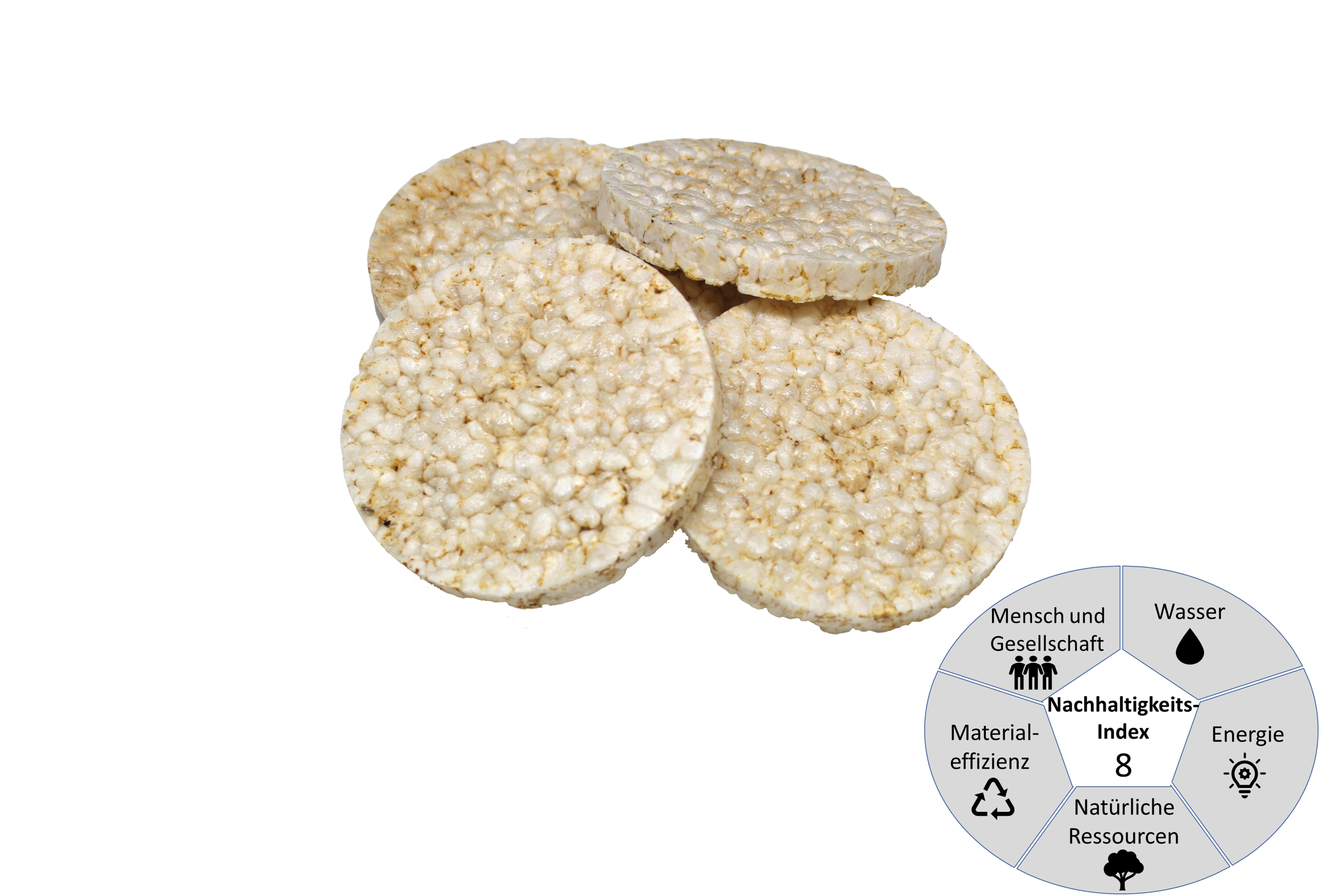


Low sustainability score


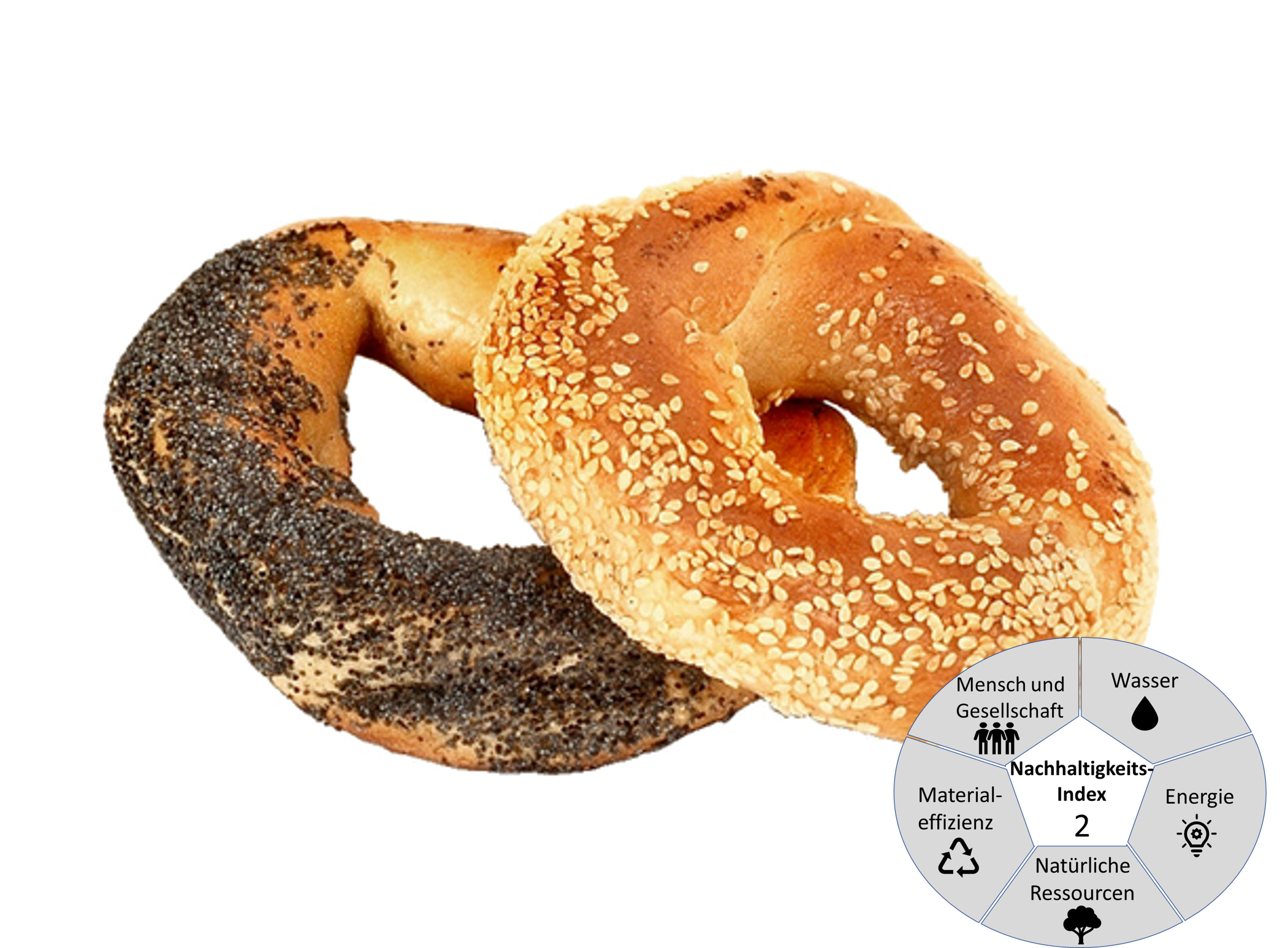

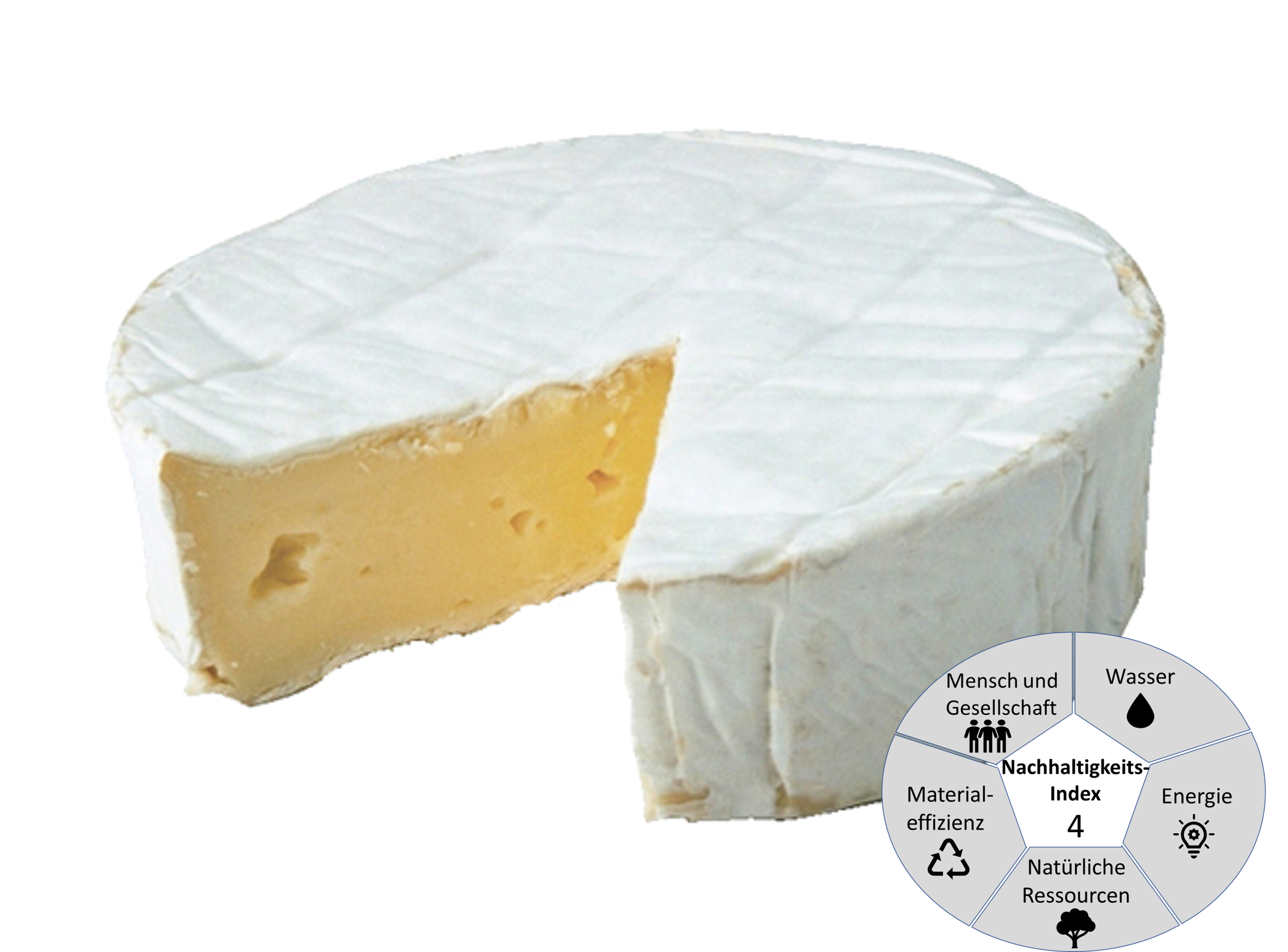

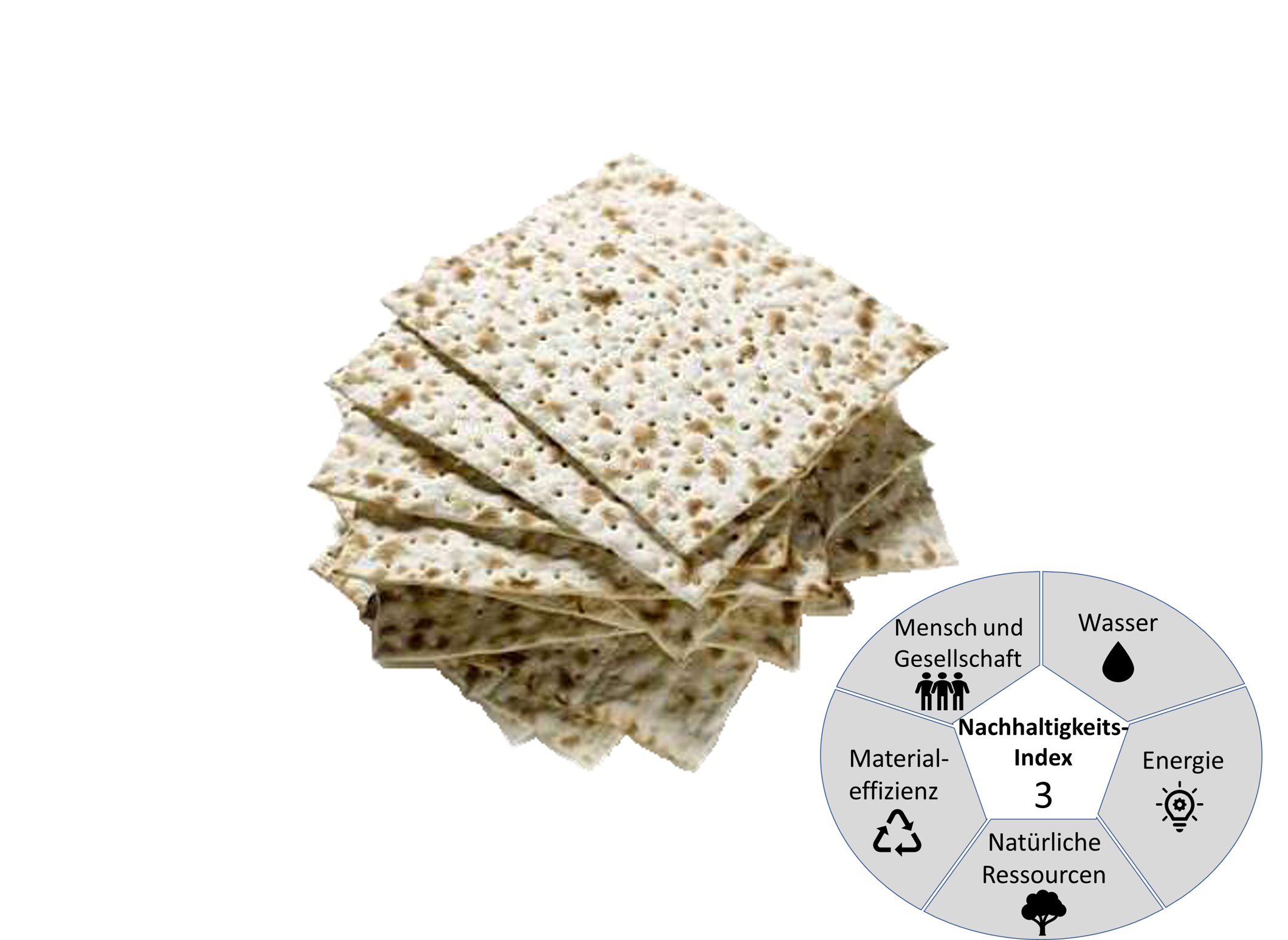

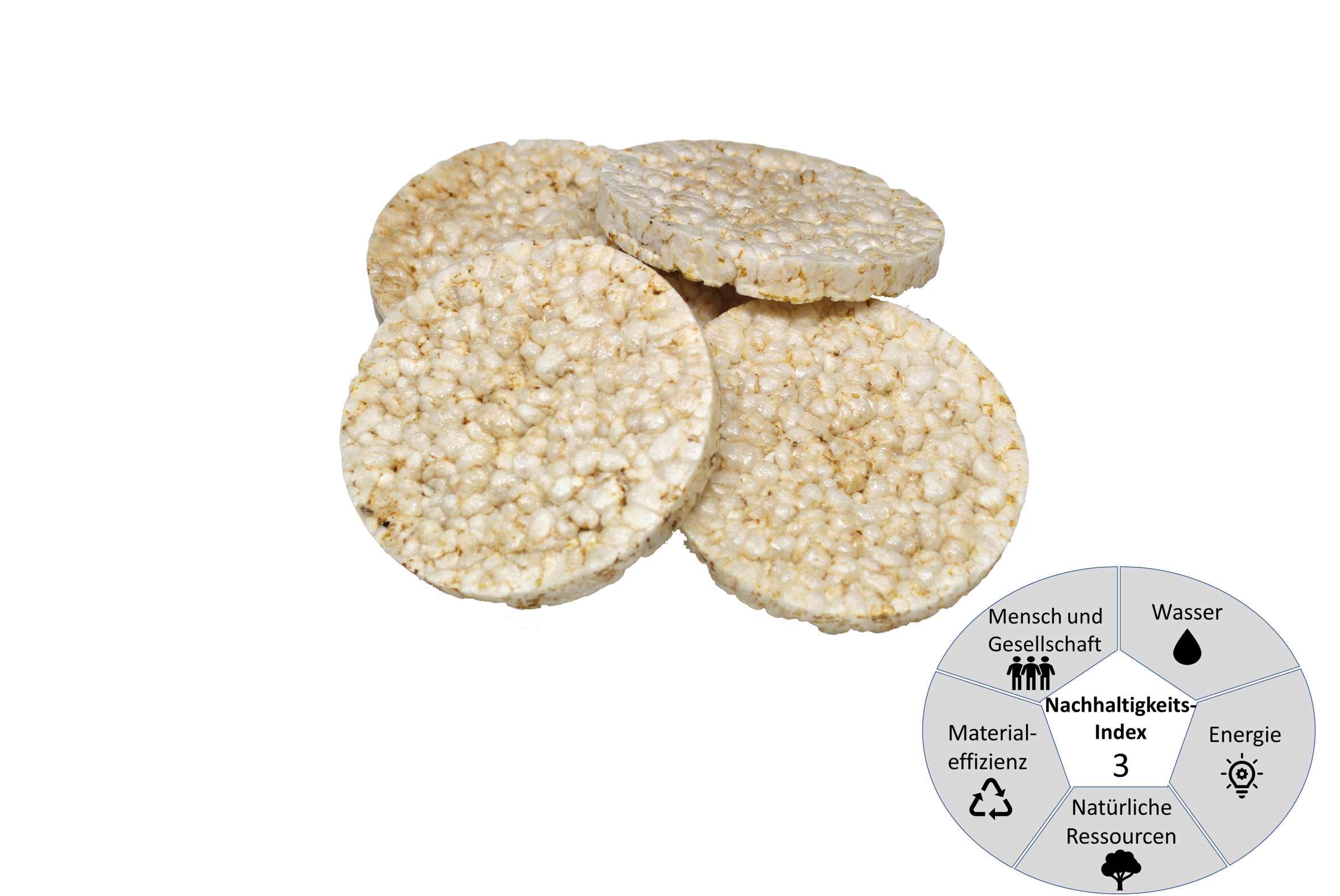


**Questionnaires**

**German version of the ‘General Health Interest’ scale (Roininen et al., 1999)**

**Wie gesund Lebensmittel sind, hat wenig Einfluss auf meine Essensentscheidungen.**The healthiness of food has little impact on my food choices. **Ich nehme es sehr genau, wie gesund die Lebensmittel sind, die ich esse.**I am very particular about the healthiness of food I eat. **Ich esse, was ich mag, und ich mache mir keine Sorgen darum, wie gesund Lebensmittel sind.**I eat what I like, and I do not worry much about the healthiness of food**.
Es ist mir wichtig, dass meine Ernährung fettarm ist.**It is important for me that my diet is low in fat**.
Ich ernähre mich immer gesund und ausgewogen.**I always follow a healthy and balanced diet**.
Es ist mir wichtig, dass meine tägliche Ernährung viele Vitamine und Mineralien enthält:**It is important to me that my daily diet contains a lot of vitamins and minerals.
**Ob ein Snack gesund ist oder nicht, macht für mich keinen Unterschied.**The Healthiness of Snacks makes no difference to me**.
Ich vermeide keine Lebensmittel, selbst wenn sie meinen Cholesterinspiegel erhöhen.**I do not avoid foods, even if they raise my cholesterol.

**‘General Health Interest’ scale (Roininen et al., 1999) adapted for sustainability**

**Wie nachhaltig Lebensmittel sind, hat wenig Einfluss auf meine Essensentscheidungen.**The sustainability of food has little impact on my food choices. **Ich nehme es sehr genau, wie nachhaltig die Lebensmittel sind, die ich esse.**I am very particular about the sustainability of food I eat. **Ich esse, was ich mag, und ich mache mir keine Sorgen darum, wie nachhaltig Lebensmittel sind.**I eat what I like, and I do not worry much about the sustainability of food**.
Es ist mir wichtig, dass meine Ernährung einen niedrigen CO2-Ausstoß verursacht.**It is important for me that my diet has a low carbon foodprint. **Ich ernähre mich immer nachhaltig und umweltbewusst.**I always follow a sustainable and environmentally conscious diet.  **Es ist mir wichtig, dass meine tägliche Ernährung überwiegend pflanzlich ist.**It is important to me that my daily diet is mostly plant-based. **Ob ein Snack nachhaltig ist oder nicht, macht für mich keinen Unterschied.**The Sustainability of Snacks makes no difference to me. **Ich vermeide keine Lebensmittel, selbst wenn sie schädlich für die Umwelt sind.**I do not avoid foods, even if they are harmful for the environment.

**Control analyses for effect of food type**

**sTable 1**

*The effect of health score and food type on sustainability ratings*

| Predictor | *df_Num_* | *df_Den_* | *F* | *p* |
| --- | --- | --- | --- | --- |
| Health | 1 | 710 | 27.82 | <.001* |
| Food | 2.87 | 2036.36 | 10.56 | <.001* |
| Health X Food | 2.87 | 2036.36 | 2.22 | .087 |

*Note.* Analysis of variance. *df_Num_* = Numerator degrees of freedom. *df_Den_* = Denominator degrees of freedom. * indicates significance.

**sTable 2**

*Bonferroni-corrected post hoc tests for the effect of health score and food type on sustainability ratings*

| Health Score | Food 1 | Mean (sd) | Food 2 | Mean (sd) | p | P adj. |
| --- | --- | --- | --- | --- | --- | --- |
| High | Cerealbar | 6.12 (2.15) | Pancakes | 5.57 (2.05) | <.001* | .005* |
| High | Cerealbar | 6.12 (2.15) | Pasta | 5.97 (1.96) | .378 | - |
| High | Pancakes | 5.57 (2.05) | Pasta | 5.97 (1.96) | .014* | .082 |
| High | Cerealbar | 6.12 (2.15) | Peanuts | 6.06 (2.39) | .71 | - |
| High | Pancakes | 5.57 (2.05) | Peanuts | 6.06 (2.39) | .003* | .018* |
| High | Pasta | 5.97 (1.96) | Peanuts | 6.06 (2.39) | .609 | - |
| Low | Cerealbar | 5.28 (2.40) | Pancakes | 5.04 (2.19) | .159 | .956 |
| Low | Cerealbar | 5.28 (2.40) | Pasta | 5.41 (2.03) | .446 | - |
| Low | Pancakes | 5.04 (2.19) | Pasta | 5.41 (2.03) | .030* | .181 |
| Low | Cerealbar | 5.28 (2.40) | Peanuts | 5.21 (2.34) | .691 | - |
| Low | Pancakes | 5.04 (2.19) | Peanuts | 5.21 (2.34) | .312 | - |
| Low | Pasta | 5.41 (2.03) | Peanuts | 5.21 (2.34) | .246 | - |

*Note.* Mean, standard deviations and uncorrected and Bonferroni-corrected p-values for the post hoc tests.

**sTable 3**

*The effect of sustainability score and food type on healthiness ratings*

| Predictor | *df_Num_* | *df_Den_* | *F* | *p* |
| --- | --- | --- | --- | --- |
| Sustainability | 1 | 710 | 9.35 | .002* |
| Food | 2.88 | 2045.41 | 67.49 | <.001* |
| Sustainability X Food | 2.88 | 2045.41 | 1.08 | .35 |

*Note.* Analysis of variance. *df_Num_* = Numerator degrees of freedom. *df_Den_* = Denominator degrees of freedom. * indicates significance.

Roininen, K., Lähteenmäki, L., & Tuorila, H. (1999). Quantification of Consumer Attitudes to Health and Hedonic Characteristics of Foods. *Appetite*, *33*(1), 71–88. https://doi.org/10.1006/appe.1999.0232
